# Mechanism Underlying the Reversal of Drug Resistance in P-Glycoprotein-Expressing Leukemia Cells by Pinoresinol and the Study of a Derivative

**DOI:** 10.3389/fphar.2017.00205

**Published:** 2017-04-25

**Authors:** María L. González, D. Mariano A. Vera, Jerónimo Laiolo, Mariana B. Joray, Mariana Maccioni, Sara M. Palacios, Gabriela Molina, Priscila A. Lanza, Samanta Gancedo, Vivian Rumjanek, María C. Carpinella

**Affiliations:** ^1^Fine Chemical and Natural Products Laboratory, School of Chemistry, Catholic University of CórdobaCórdoba, Argentina; ^2^Department of Chemistry, QUIAMM–INBIOTEC–CONICET, College of Exact and Natural Sciences, National University of Mar del PlataMar del Plata, Argentina; ^3^Immunology, Department of Biochemical Chemistry, CIBICI-CONICET, School of Chemical Sciences, National University of CórdobaCórdoba, Argentina; ^4^Institute of Medical Biochemistry, Federal University of Rio de JaneiroRio de Janeiro, Brazil

**Keywords:** multidrug resistance reversal, P-glycoprotein, plant-derived compounds, (±) pinoresinol, 1-acetoxy-(+)-pinoresinol

## Abstract

P-glycoprotein (P-gp) is a membrane protein associated with multidrug resistance (MDR) due to its key role in mediating the traffic of chemotherapeutic drugs outside cancer cells, leading to a cellular response that hinders efforts toward successful therapy. With the aim of finding agents that circumvent the MDR phenotype mediated by P-gp, 15 compounds isolated from native and naturalized plants of Argentina were screened. Among these, the non-cytotoxic lignan (±) pinoresinol successfully restored sensitivity to doxorubicin from 7 μM in the P-gp overexpressed human myelogenous leukemia cells, Lucena 1. This resistance-reversing effect was confirmed by competitively increasing the intracellular doxorubicin accumulation and by significantly inhibiting the efflux of doxorubicin and, to a lesser extent, that of rhodamine 123. The activity obtained was similar to that observed with verapamil. No such results were observed in the sensitive parental K562 cell line. To gain deeper insight into the mode of action of pinoresinol, its effect on P-gp function and expression was examined. The docking simulations indicated that the lignan bound to P-gp at the apex of the V-shaped transmembrane cavity, involving transmembrane helices 4, 5, and 6, and partially overlapped the binding region of tariquidar, which was used as a positive control. These results would shed some light on the nature of its interaction with P-gp at molecular level and merit further mechanistic and kinetic studies. In addition, it showed a maximum 29% activation of ATP hydrolysis and antagonized verapamil-stimulated ATPase activity with an IC_50_ of 20.9 μM. On the other hand, pinoresinol decreased the presence of P-gp in the cell surface. Derivatives of pinoresinol with improved activity were identified by docking studies. The most promising one, the non-cytotoxic 1-acetoxypinoresinol, caused a reversion of doxorubicin resistance from 0.11 μM and thus higher activity than the lead compound. It also caused a significant increase in doxorubicin accumulation. Results were similar to those observed with verapamil. The results obtained positioned these compounds as potential candidates for effective agents to overcome P-gp-mediated MDR, leading to better outcomes for leukemia chemotherapy.

## Introduction

The P-glycoprotein (P-gp) transporter (ABCB1/MDR1) encoded by the *MDR-1* gene is a protein located in the cell membranes of various tissues involved in the traffic of substrates outside the cells (Sharom, 2011; Silva et al., 2015). In a considerable number of cancers, high levels of *MDR-1* expression provide the most commonly encountered mechanism of multidrug resistance (MDR) (Shin et al., 2006), representing a major obstacle to the success of chemotherapy (Siarheyeva et al., 2010).

P-glycoprotein (P-gp) is comprised of two homologous transmembrane domains (TMDs). Each half consists of six transmembrane α-helices (TMHs) and one cytoplasmic nucleotide-binding domain (NBD), which fuel the energy from ATP hydrolysis, leading to conformational changes that result in the extrusion of a set of structurally and functionally unrelated chemotherapy drugs against their concentration gradient (Sharom, 2011). As a consequence, P-gp keeps intracellular drug accumulation low, leading to a cellular responsiveness known as classical MDR (Krishna and Mayer, 2000). This phenomenon and the broad spectrum of substrates removed from cells, such as paclitaxel, etoposide, teniposide, vinblastine, vincristine, doxorubicin, daunorubicin, and imatinib among others (Kathawala et al., 2015), makes this pump one of the most significant transporters in pharmacology (Saeed M. E. M. et al., 2015).

Almost half of human tumors show the ability to express P-gp (Fu and Arias, 2012). Not only failures in chemotherapy but also poor overall prognosis are strongly linked to increased levels of the *MDR-1* product in many cancers (Loo and Clarke, 1999), including certain types of leukemia (Szakacs et al., 2006; Vasconcelos et al., 2011; Rumjanek et al., 2013). Leukemia is a malignant disorder with a significant number of deaths annually (Lin et al., 2011). Based on GLOBOCAN, about 352,000 new cases of leukemia and 265,000 deaths occurred worldwide in 2012 (Ferlay et al., 2015). Overexpression of P-gp was detected in about 50% of patients with chronic myelogenous leukemia (CML) unresponsive to chemotherapy with Vinca alkaloids and anthracyclines (Kuwazuru et al., 1990).

Strategies to overcome MDR include the development of P-gp function inhibitors that may act by blocking the substrate binding to the protein, by interacting with an allosteric region of P-gp preventing the efflux or by interfering with the ATP hydrolysis. Alternatively, inhibitors may act by indirect mechanisms, like hindering P-gp phosphorylation or disturbing the integrity of the cell membrane lipids (Wink, 2012; Silva et al., 2015). Interference with the surface expression of P-gp is proposed as another valid strategy for restoring drug effectiveness (Ferrándiz-Huertas et al., 2011; Fu and Arias, 2012).

Although some of the reversal agents submitted to clinical trials succeed in some patients (List et al., 2001), most of them failed in many aspects to prove their effectiveness as MDR reversers (Wink, 2012; Lei et al., 2013), particularly in their adverse effects, interactions with the drug administered in parallel and inadequate trial designs (List et al., 2001; Szakacs et al., 2006; Steinbach and Legrand, 2007; Wu et al., 2011; Xia et al., 2015).

A great deal of research therefore focuses on the search for agents devoid of these undesired effects and able to reverse the MDR/P-gp phenotype.

Plants constitute an important source of bioactive molecules with a significant contribution to cancer chemotherapy (Gosh et al., 2009) including P-gp inhibitors (Palmeira et al., 2012a). The exceptional structural diversity of plant-derived metabolites offers a great range of possibilities for finding novel modulators of this target. As observed with substrates, P-gp chemosensitizers can be structurally distinct (Eid et al., 2015), with a diversity of plant compounds belonging to different chemical families that are capable of suppressing P-gp-mediated transport (Efferth et al., 2002; Katayama et al., 2007; Nabekura et al., 2008; Han et al., 2011; Wink, 2012; Eid et al., 2013; Lei et al., 2013; Sun and Wink, 2014; Zeino et al., 2015). These entities, together with compounds from other natural sources, are known as fourth-generation inhibitors (Wu et al., 2011).

Even though many compounds with medicinal properties have been obtained from Argentine flora (Chiari et al., 2010, 2011; Carpinella et al., 2011; Joray et al., 2011, 2013), this resource is far from being completely explored, especially for compounds with MDR reversal properties. It is considered that only 1% of the 9,690 species of Argentine vascular flora have been studied. Among these species, mainly belonging to Asteraceae, Poaceae and Fabaceae, 1,200 are known to possess medicinal properties (Zuloaga et al., 1999; Alonso and Desmarchelier, 2015).

With this in mind, we screened a panel of bioactive metabolites obtained from native and naturalized plants from central Argentina on P-gp overexpressed leukemia cells. Assays were performed focused on the modes of action of the most effective compound, pinoresinol, including studies of the binding site of this active principle on P-gp by molecular modeling. The lignan (±)-pinoresinol was previously isolated in our laboratory from the naturalized tree, *Melia azedarach* as an antifungal compound (Carpinella et al., 2003). It was able to decrease the effective concentrations of the synthetic antifungal agents mancozeb and carboxin, even when these were applied at 5 and 3% of their corresponding minimum inhibitory concentrations (Carpinella et al., 2005). Pinoresinol also showed anti-inflammatory, antioxidant, neuroprotective, and hypoglycemic properties (López-Biedma et al., 2016) and exhibited different levels of cytotoxic effect depending on the tumor cells (Moon et al., 2008; López-Biedma et al., 2016). Few *in vivo* studies have been performed and these have concentrated on the anti-inflammatory, antioxidant, antitumor, and neuroprotective effect of the lignan (Torres-Sanchez et al., 2009; Kim et al., 2010; Lapi et al., 2015).

In addition, a pinoresinol derivative, showing improved activity in relation to pinoresinol in docking simulations, showed experimentally an outstanding reversing activity on P-gp transport, with higher effectiveness than the lead compound.

## Materials and methods

### Chemicals, equipment, and reagents

3-(4,5-Dimethyl-2-thiazolyl)-2,5-diphenyl-2H-tetrazolium bromide (MTT), vinblastine sulfate (VLB, 96%), cyclosporine A (CsA, 98.5%), trifluoperazine dihydrochloride (TFP, 99.0%), histopaque 1077, rhodamine 123 (Rho 123), and lectin from *Phaseolus vulgaris* (PHA) were purchased from Sigma-Aldrich Co (St Louis, MO). Doxorubicin hydrochloride (DOX, 99.8%, Synbias Pharma Ltd.) was obtained from Nanox Release Technology (Buenos Aires, Argentina) and was used dissolved in bi-distilled water. Verapamil hydrochloride 98% was provided by Parafarm (Buenos Aires, Argentina) and was used as the reference P-gp inhibitor dissolved in ethanol at 30 μM. RPMI-1640 and Gibco® cell culture reagents were purchased from Invitrogen Life Technologies (Carlsbad, CA). Sterile plastic material was purchased from Greiner Bio-One (Frickenhausen, Germany). All solvents were HPLC grade.

FITC mouse anti-human (P-gp) was purchased from BD (BD Biosciences, USA). MDR1 PREDEASY™ ATPase assay kit was obtained from Solvo Biotechnology (Szeged, Hungary). Flow cytometry was performed in a Becton Dickinson (BD) FACSCanto II flow cytometer (BD Biosciences, USA).

**Figure 1:**
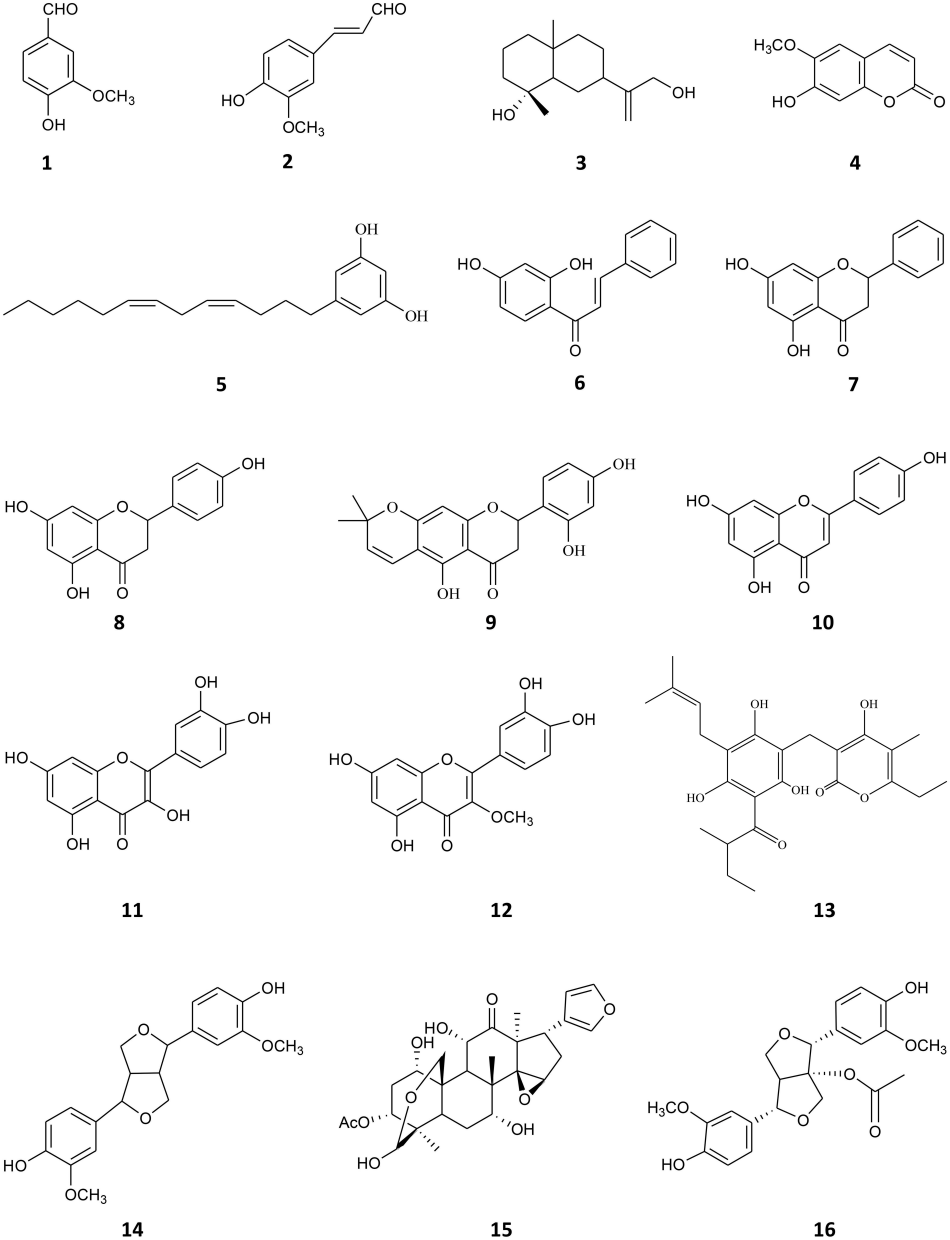
**Chemical structures of compounds 1–16**.

Compounds **1–15** (Figure 1) were previously isolated in the laboratory from native and naturalized plants from Argentina. They were tested at 90% or higher purity, determined by HPLC. The compounds assayed were: vanillin (**1**) and, 4-hydroxy-3-methoxycinnamaldehyde (**2**), both isolated from *Melia azedarach* (Carpinella et al., 2003), ilicol (**3**) isolated from *Flourensia oolepis* (Diaz Napal and Palacios, 2013), scopoletin (**4**) obtained from *M. azedarach* (Carpinella et al., 2005), (*Z,Z*)-5-(trideca-4,7-dienyl)-resorcinol (**5**) isolated from *Lithrea molleoides* (Carpinella et al., 2011), 2′,4′-dihydroxychalcone (**6**) and (-)-pinocembrin (**7**) both obtained from *F. oolepis* (Diaz Napal et al., 2009; Joray et al., 2015), naringenin (**8**) isolated from *Baccharis salicifolia* (Céspedes et al., 2006; del Corral et al., 2012), dalenin (**9**) obtained from *Dalea elegans* (Chiari et al., 2011), apigenin (**10**) isolated from *B. salicifolia* (del Corral et al., 2012), quercetin (**11**), 3-*O*-methylquercetin (**12**), and 23-methyl-6-*O*-desmethylauricepirone (**13**) obtained from *Achyrocline satureioides* (Joray et al., 2011, 2013) and (±)-pinoresinol (**14**) and meliartenin (**15**) both obtained from *M. azedarach* (Carpinella et al., 2002, 2003). 1-acetoxy-(+)-pinoresinol (**16**) (Figure 1) was purchased from Chem Faces (Wuhan, PR). The ^1^H, ^13^C, 2D-NMR spectra, and HPLC chromatograms of the compounds are available upon request from the authors.

### Cell lines and cell culture

**Figure 2:**
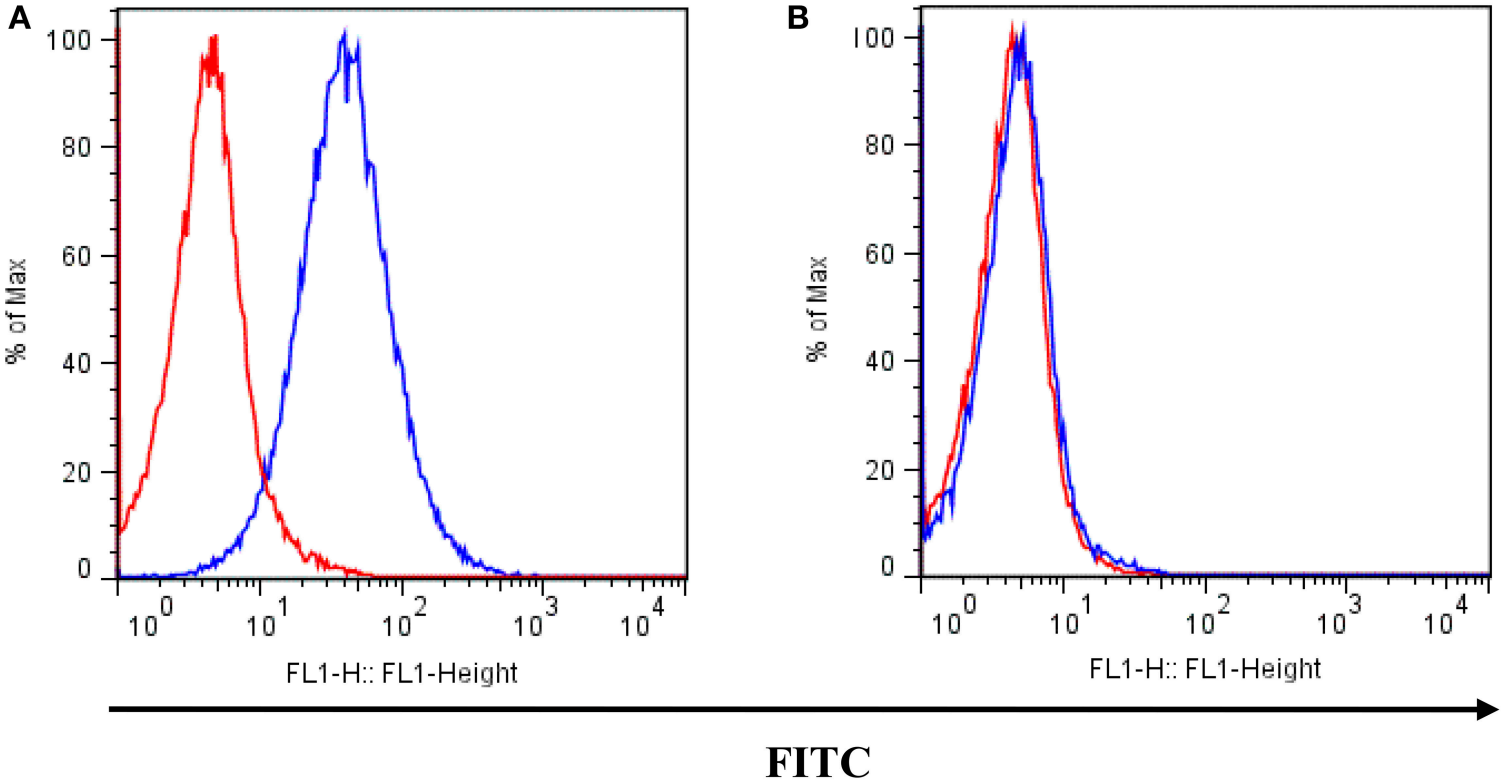
**P-gp surface expression in (A)** Lucena 1 and **(B)** K562 cells determined by flow cytometry. Unstained cells are shown with red histogram while FITC-conjugated mouse anti-human P-gp antibody stained cells are shown with blue histogram. Histograms are representative of three independent experiments.

The K562 human CML cell line (Rumjanek et al., 2013) and its MDR counterpart, Lucena 1, were used (Moreira et al., 2014). Lucena 1 was chosen as a well-characterized resistant cell line in which P-gp overexpression is the exclusive mechanism of acquired resistance (Moreira et al., 2014). Real time quantitative PCR analysis for P-gp and MRP1 showed no statistical differences between cells maintained with vincristine and those maintained with doxorubicin. Both lines were routinely maintained in RPMI-1640 medium supplemented with 10% fetal bovine serum, 2 mM L-glutamine, 100 U mL^−1^ penicillin and 100 μg mL^−1^ streptomycin in a 5% CO_2_ humidified atmosphere at 37°C. Cells were subcultured twice a week and used when under 20th passage from frozen stocks. Lucena 1 cells were cultured in the presence of 60 nM of DOX to maintain P-gp overexpression, until 4 days before the experiments, when they were transferred to drug-free medium. These cells displayed a higher superficial P-gp expression than parental K562 cells (see Figure 2). All experiments were performed with the cells in the logarithmic growth phase with cell viabilities above 90% determined by staining with trypan blue.

### Multidrug resistance reversal assay

Compounds **1–15** and then compound **16**, which was selected after demonstrating lower binding energy than its lead compound **14** and other pinoresinol derivatives, were screened by MTT colorimetric assay for their ability to reverse cellular resistance to DOX. Briefly, 5 × 10^4^ Lucena 1 cells per well were seeded in 96-well plates containing RPMI-1640 medium in the presence of DOX alone or in combination with the tested compounds dissolved in ethanol or acetonitrile as appropriate. DOX was added to reach final concentrations of 0.05–431 μM. The compounds were assayed at the highest non-toxic concentration observed in both cell lines (cytotoxicity ≤ 20%, Cytotoxicity (%) = [1-(Optical density treatment-Optical density DMSO)/(Optical density control-Optical density DMSO)] x 100, determined by MTT proliferation assay (Joray et al., 2015); maximum concentration tested 40 μg mL^−1^). Following the primary screening, compounds **14** and **16** (dissolved in ethanol) were tested at serial dilutions. Negative controls containing only bi-distilled water, ethanol or acetonitrile (1% v/v since no adverse effects were observed at this concentration) were simultaneously run as well as viability controls with no addition of the dissolution solvents. Verapamil was used as a positive control at 30, 0.11 and 0.055 μM. After 48 h incubation at 37°C with 5% CO_2_, 20 μL of 5 mg mL^−1^ MTT solution in sterile PBS was added to each well and incubation continued for 4 h. Subsequently, the supernatants were removed and replaced with 100 μL DMSO to solubilize the resulting purple formazan crystals produced from metabolically viable cells. Absorbance was measured with an iMark micro-plate reader (Bio-Rad, USA) at 595 nm.

Half inhibitory concentrations (IC_50_) represent the concentrations of DOX required to inhibit 50% of cell proliferation (compared to the solvent controls, which showed no differences in comparison to viability controls) and were calculated from the mean values of data from wells.

The same reversal assay was performed in K562 cells, in order to discard a decrease in the IC_50_ of DOX due to effects other than P-gp inhibition. The reversal fold (RF) values for the tested compounds, which indicate their capacity to reduce resistance to DOX, were calculated by dividing the IC_50_ of DOX alone by the IC_50_ of DOX in the presence of the tested compounds (Xu et al., 2014).

The assays were also performed with VLB, which was added, dissolved in DMSO at 2 × 10^−5^ to 44 μM alone or in combination with **14** at 112 μM. The negative control contained DMSO, while verapamil was used as the reference compound.

### Doxorubicin intracellular accumulation assays

The effect of **14** or **16** on DOX intracellular accumulation was further studied.

In order to establish the best assay conditions, 2.5 × 10^5^ Lucena 1 or K562 cells mL^−1^ were seeded onto 24-well plates and pre-incubated in complete RPMI-1640 medium in the presence of 112 μM of **14** (dissolved in ethanol), verapamil or ethanol (1% v/v) from 0 to 48 h at 37°C with 5% CO_2_. Following incubation, 5 μM DOX was added and cells were further incubated for 1 h.

Time course of DOX accumulation was investigated by co-incubating 2.5 × 10^5^ Lucena 1 or K562 cells mL^−1^ with 112 μM of **14** or 1% ethanol in 96-well plates for 1 h at 37°C prior to the addition of 5 μM DOX. The intracellular DOX was measured at increasing periods of time.

To obtain further information about the type of inhibition exerted by compound **14**, the kinetic behavior was analyzed by the Lineweaver-Burk double-reciprocal method compared to data obtained in the absence of the inhibitor (ethanol control) (Arnaud et al., 2010). The drug retention rate corresponding to the amount of DOX remaining inside 50,000 Lucena 1 cells after 1 h incubation was plotted vs. DOX concentrations and fitted with the Michaelis-Menten equation, R = R_max_ [S]/K_m_ + [S], where R is the drug retention rate, R_max_ the maximal drug retention rate, [S] is the substrate concentration and K_m_ the Michaelis constant of DOX efflux (Copeland, 2000; Arnaud et al., 2010).

With the aim of determining the minimum effective concentration (MEC) of **14** or **16**, 2.5 × 10^5^ Lucena 1 or K562 cells mL^−1^ were incubated with 7–112 or 0.027–14 μM of each compound, respectively, for 1 h at 37°C with 5% CO_2_. Afterwards, 5 μM solution of DOX was added and the plates were further incubated for 1 h. Verapamil or ethanol (1%) were run as positive and negative controls, respectively. Viability controls were included in all the experiments.

After DOX incubation in all the assays described, cells were placed on ice to stop the reactions, followed by washing twice with ice-cold PBS. The intracellular DOX-associated fluorescence of 50,000 individual cells was determined by FACScan flow cytometry. DOX was excited at 488 nm and the emitted light was collected with a 585/42 nm bandpass filter. Dead cells and cell debris were excluded by forward and side scatter gating. Mean fluorescence intensities (MFI) were analyzed with Flowjo software (Tree Star, Inc. Ashland, OR).

The data collected was compared to that obtained with 30 μM verapamil (which was considered as the maximum inhibition) and expressed as the published equation (Huang et al., 2013):

$$\begin{array}{l} \%\text{ of Inhibition}\;=\;[\frac{\left(\text{MFI of cells treated with }14\text{ or }16\;-\text{ MFI of negative control}\right)}{\left(\text{MFI of cells treated with verapamil }-\text{ MFI of negative control}\right)}]\times 100 \end{array}$$

With the aim of discarding fluorescence by the compounds themselves, flow cytometry of **14** or **16** was performed at the maximum concentration tested in the absence of DOX. No fluorescence due to these compounds was observed.

### Doxorubicin efflux assay

To determine the effect on DOX efflux, 2.5 × 10^5^ Lucena 1 or K562 cells mL^−1^ were first pre-incubated in 24-well plates in the presence of **14**, dissolved in ethanol at 112 μM, verapamil or ethanol (1% v/v) for 1 h at 37°C with 5% CO_2_. After incubation, DOX 5 μM was added to the reaction mixture and further incubated for 1 h. Subsequently, cells were washed with cold PBS and incubated for different times with **14**, verapamil or ethanol in DOX-free medium to allow dye extrusion. With the aim of determining the minimum effective concentration (MEC) of **14**, 2.5 × 10^5^ Lucena 1 cells were incubated with 7–112 μM of the lignan, for 1 h at 37°C with 5% CO_2_. Afterwards, 5 μM solution of DOX was added and the plates were further incubated for 1 h. After washing, cells were incubated for 30 min with **14**. Verapamil or ethanol (1%) were run as positive or negative controls, respectively. Cells were then washed with ice-cold PBS and the intracellular DOX-associated MFI of 50,000 individual cells was determined by flow cytometry as previously described.

### Rhodamine 123 efflux assay

Rho 123, a P-gp fluorescent substrate, is frequently employed as an indicator of P-gp activity and it was therefore used in an additional efflux study. Briefly, Lucena 1 or K562 cells at a density of 2.5 × 10^5^ mL^−1^ were pre-incubated with **14** dissolved in ethanol at 112 μM (2 × MEC), TFP (8 μM), verapamil or ethanol (1% v/v) for 1 h at 37°C with 5% CO_2_. Subsequently, cells were incubated with 500 ng mL^−1^ of Rho 123 for 30 min. After incubation, the medium was removed and the cells were incubated in Rho 123-free medium with **14**, verapamil, TFP and ethanol, for a further 30 min to allow Rho 123 extrusion. After washing twice with cold PBS, the amount of Rho 123 remaining in 10,000 individual cells after the efflux period was quantified by FACScan flow cytometry. Excitation was performed with a laser operating at 488 nm and the emitted fluorescence was collected through a 530/30 nm pass filter.

### Determination of ATPase activity

The ATPase activity of P-gp was determined using the PREDEASY™ ATPase assay kit as per the manufacturer's recommendation. In the activation assay, increasing concentrations of **14** (0.27–600 μM) or verapamil (0.14–300 μM), both dissolved in DMSO (2% final concentration), were pre-incubated in ATPase assay buffer with membrane preparations from *Spodoptera frugiperda* ovarian cells (Sf9) containing human P-gp and 10 mM MgATP for 10 min at 37°C. For monitoring the inhibition of the substrate stimulated-ATPase activity, verapamil (40 μM) was added to the incubation mixture as an ABC transporter-related ATPase activator. The ATPase reaction was subsequently stopped and the inorganic phosphate (Pi) produced was measured colorimetrically at 630 nm. DMSO was used as a solvent control and CsA (final concentration of 40 μM) was used as reference inhibitor in the inhibition assay.

All experiments were performed in the absence or presence of 1.2 mM of sodium orthovanadate, an ABC transporter-related ATPase inhibitor, in order to measure the vanadate-sensitive portion of the total ATPase activity. ATPase activities were determined as Activity (%) = (A − B) – (E − F) × 100/(C − D) – (E − F), where A is the activity in the presence of **14** alone or with verapamil as activator in the inhibition study, B is the activity in the presence of **14** alone or with verapamil in the inhibition study, in the presence of vanadate (background activity), C is the maximum activation value due to verapamil and D is the same value as C but with the addition of vanadate (background activity), E is the activity of control with DMSO while F is the activity of DMSO in the presence of vanadate. Membranes from Sf9 cells expressing defective P-gp were used as controls.

### Molecular modeling

The structural model of the P-gp used for the docking studies was a homology model of the human P-gp previously proposed in Jara et al. (2013). In this work, this model was subject to stochastic molecular dynamics for obtaining average inter-residue distances in good agreement with the experimental distances and challenged to reproduce the right activity order of a pool of known inhibitors, as well as to find binding sites in good agreement with those proposed on computational and experimental bases in the literature (Jara et al., 2013).

The following probe ligands were docked in the structural models of the P-gp:

- pinoresinol derivatives, such as 1-acetoxy-(+)-pinoresinol (**16**), 1-acetoxy-(−)-pinoresinol (**16a**), 1-hydroxy-(+)-pinoresinol, phylligenin, pinoresinol diglucoside and (+)-pinoresinol 4-glucoside submitted to docking to establish whether their activity improved with respect to **14**.
- compound **2**, which was found to be inactive, was used as a negative control and the powerful inhibitor tariquidar (XR9570) (Jara et al., 2013) run as positive control, even though it was not experimentally studied.
- Verapamil, a known inhibitor used as a reference in the experiments.
- DOX and Rho 123 used as model substrates in the experiments.

The structures of these ligands were obtained by performing a conformational search (when relevant) and a full geometry optimization at the semiempirical PM6 level of theory, characterizing the structures as minima by diagonalizing the Hessian matrix and ensuring the absence of negative eigenvalues; next, a refinement was made at the PBE0/6-31G^*^ level using the Gaussian 09 (Rev. B01) package (http://www.gaussian.com). The Autodock 4.2.6 package (Morris et al., 2009) was used for the docking simulations, precomputing a grid in the interior of the whole TMD. Considering the large size of the docking region, 2,000 runs of Lamarckian genetic algorithm were performed for each ligand (Morris et al., 1998, 2009). The population was set at 150 individuals, up to 10^5^ generations with 1 survivor per generation and a limit of 6 × 10^6^ energy evaluations and the remaining algorithm control parameters set to program defaults. The cluster analysis was made with 2.5Å of RMSD. Molecular graphics rendering was performed using VMD 1.9.2 (Humphrey et al., 1996).

Besides the main grid box including the transmembrane region, molecular docking on the NBDs was carried out to obtain preliminary information about the presence of direct binding of **14** to the NBDs.

### Study on the surface expression of P-Gp

To determine the effect of **14** on P-gp surface expression, Lucena 1 or K562 cells at a density of 5 × 10^4^ cells mL^−1^ were incubated in the presence of **14**, dissolved in ethanol at 112 μM (maximum non-cytotoxic concentration, in order to ensure the presence of the effect) or 1% ethanol (control) for 24 and 48 h. Then, cells were washed with cold PBS and labeled for 30 min at 4°C in the dark with FITC-conjugated mouse anti-human P-gp antibody, which binds to an external epitope of P-gp, according to the manufacturer's instructions. Finally, cells were washed and suspended with ice-cold PBS and fluorescence intensity was determined in 10,000 individual cells by FACScan flow cytometry. FITC fluorescence was measured at an excitation wavelength of 488 nm and emitted light was collected with a 530/30 nm bandpass filter.

### Cytotoxicity on peripheral blood mononuclear cells (PBMC)

The cytotoxicity of **14** and **16** on peripheral blood mononuclear cells (PBMC) was evaluated by MTT assay (Joray et al., 2015). PBMC were collected from fresh heparinized blood and separated by density gradient centrifugation (Ficoll®) as described by Rennó et al. (2008). As the current study required samples from healthy human volunteer donors, ethical approval was provided by the Catholic University of Córdoba Research Ethics Board. Signed informed consents were obtained from donors. For the cytotoxicity assay, 1 × 10^5^ PBMC/well were incubated in duplicate in 96-well plates with PHA 10 μg mL^−1^, in the presence of increasing concentrations of **14** (28–560 μM), **16** (3.5–315 μM) (both dissolved in ethanol) or 1% ethanol for 48 h. Absorbance (Abs) and percentage of cytotoxicity were determined as described above and the IC_50_ values were calculated. The experiment was carried out in two separate stages.

### Statistical analysis

The results are expressed as mean ± SE. Data were analyzed using Student's *t*-test or two-way analysis of variance (ANOVA) using GraphPad Prism software (Graphpad Prism 5.0, Graphpad Software, Inc., CA, USA), with *p* ≤ 0.05 as statistically significant. All experiments were performed in duplicate or triplicate at least three times. The 50% inhibitory concentrations (IC_50_) were calculated by log-Probit analysis responding to at least seven concentrations at the 95% confidence level with upper and lower confidence limits. The curves from the ATPase assays were fitted to the relative activity vs. compound **14** concentrations plot using non-linear regression. Top (maximal response) and bottom (maximally inhibited response) values were not constrained to constant values 100 and 0, respectively.

## Results

### Multidrug resistance reversal assay

**Figure 3:**
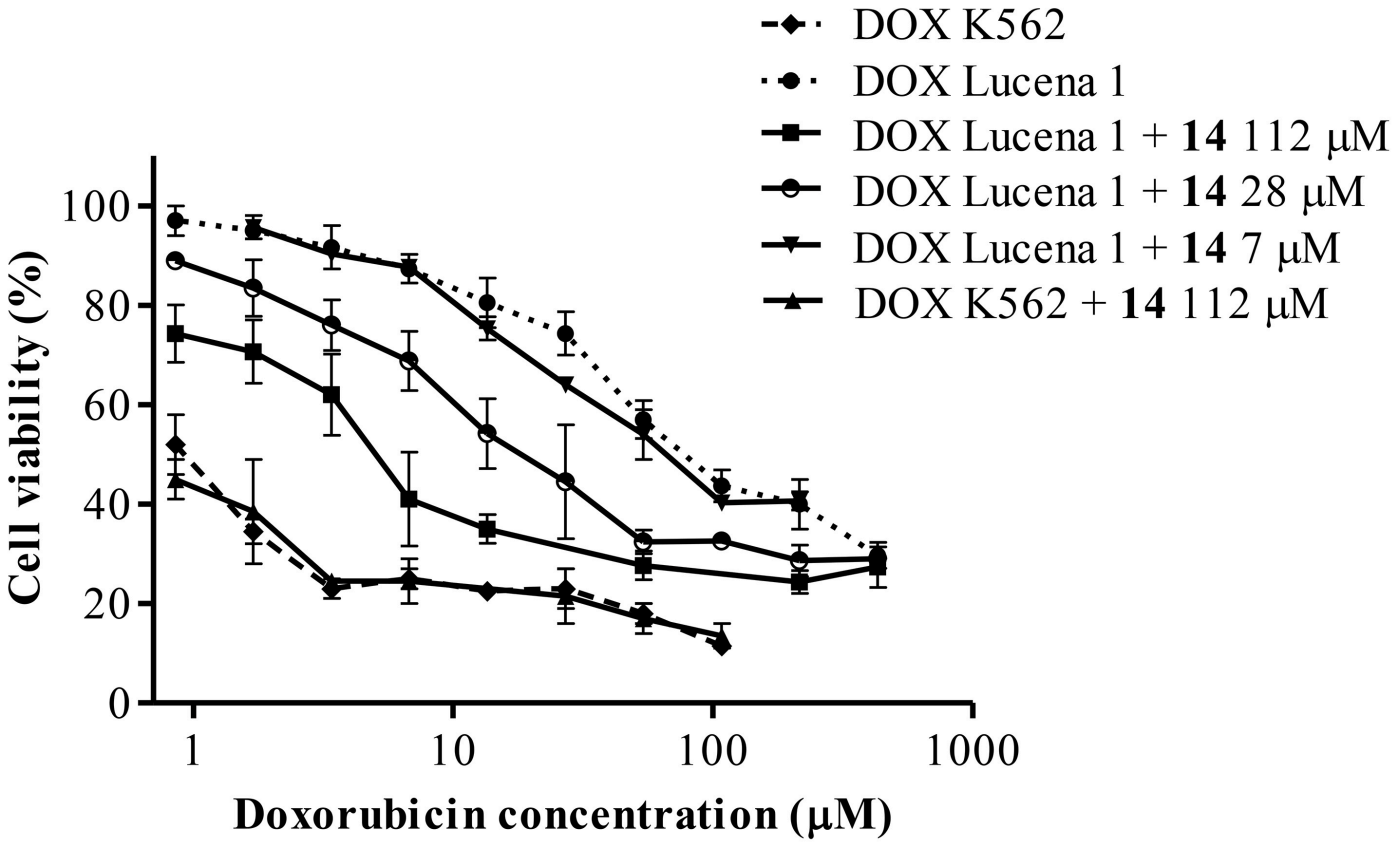
**Dose-response curves for cytotoxicity of doxorubicin (DOX) in Lucena 1 and K562 cells with and without pinoresinol (14) as determined by the resistance reversal assay**. Values are expressed as mean ± SE of at least three independent experiments.

Since there is no common pharmacophore determining that a compound behaves as a P-gp reverser (Yuan et al., 2012) and due to the wide diversity of chemical structures that interact with this transporter (Robert and Jarry, 2003) as well as the lack of information on metabolites from Argentinian flora as P-gp chemosensitizers, we decided to investigate this effect in 15 plant-derived compounds belonging to different chemical families obtained from plants from central Argentina (Carpinella et al., 2002, 2003, 2005; Diaz Napal et al., 2009; Chiari et al., 2010, 2011; Joray et al., 2011, 2013, 2015; del Corral et al., 2012; Diaz Napal and Palacios, 2013). Compounds **1–15** were studied by an MTT assay, in order to evaluate the potentiation of DOX cytotoxicity in insensitive Lucena 1 cells, which were 35-fold more resistant to this drug [IC_50_ = 40.78 (16.60–100.18) μM] than their parental cell line K562 [IC_50_ = 1.16 (0.52–2.58) μM]. The combination of the maximum non-toxic concentrations of the tested compounds with DOX showed the flavonoid **9** and the lignan **14** as the most effective, enabling the IC_50_ value of DOX in Lucena 1 cells to be decreased by a factor of 3.2 and 9.4, respectively (Table 1). The latter value is similar to that obtained with verapamil 30 μM (*p* > 0.05). When **14** was also tested at 28 μM, it caused a 3.4-fold increase in Lucena 1 sensitivity to DOX (Table 1), indicating that this compound was as active as **9** (*p* > 0.05). Given the highest effect reached by **14** when tested at a higher concentration, due to its low cytotoxicity, we selected **14** as the first compound to be further studied. As shown in Table 1, compound **14** was able to potentiate DOX toxicity in a dose-dependent manner (*b* = 0.070; *p* = 0.013; CI 95% = 0.016 to 0.124) from 7 μM (see also Figure 3). The comparison of the dose-response curves of DOX alone and in combination with **14** clearly showed the enhancement in DOX cytotoxicity (Figure 3). It is worth noting that **14** did not increase DOX sensitivity in K562 (Table 1 and Figure 3), while compounds **6** and **9** caused a decrease in DOX IC_50_, with the former also showing reversing properties in Lucena 1 (Table 1). These results further support the need to first investigate the activity of compound **14**.

**Table 1:** **Reversal effects of compounds 1-16 on P-gp mediated resistance of Lucena 1 cells**.

**Compounds (μg mL^−1^ / μM)**	**RF**	
	**K562**	**Lucena 1**
1 (40/263)	0.87 ± 0.09	0.59 ± 0.21
2 (10/56)	17.00^*^ ± 0.25	1.23 ± 0.17
3 (40/168)	0.83 ± 0.12	1.35^*^ ± 0.14
4 (40/208)	3.92^*^ ± 2.83	0.82^**^ ± 0.07
5 (10/36)	1.63 ± 1.05	0.93 ± 0.22
6 (2.5/10)	3.48^*^ ± 0.67	1.73^*^ ± 0.22
7 (20/78)	1.11 ± 0.19	0.53 ± 0.17
8 (40/147)	0.29 ± 0.08	0.68 ± 0.10
9 (10/28)	1.83^*^ ± 0.52	3.19^**^ ± 0.24
10 (20/74)	1.00 ± 0.01	0.71 ± 0.12
11 (5/17)	1.18 ± 0.18	0.47^**^ ± 0.04
12 (2.5/8)	2.17^**^ ± 0.64	0.84 ± 0.19
13 (20/45)	0.49^*^ ± 0.04	1.11 ± 0.17
14 (40/112)	1.11 ± 0.15	9.42^*^ ± 4.92
(20/56)	−	2.57^**^ ± 0.20
(10/28)	−	3.44^**^ ± 0.86
(5/14)	−	1.90^*^ ± 0.28
(2.5/7)	−	1.61^*^ ± 0.18
(1.25/3.5)	−	0.92^*^ ± 0.02
15 (0.3/0.55)	5.68^**^ ± 3.31	0.67^*^ ± 0.23
16 (12/28)	17.26^*^ ± 3.17	96.79^**^ ± 4.60
(6/14)	1.05 ± 0.14	22.66^**^ ± 2.68
(3/7)	−	5.54^*^ ± 0.59
(1.5/3.5)	−	3.49^*^ ± 0.27
(0.75 /1.75)	−	3.16^*^ ± 0.48
(0.37/0.87)	−	1.90^*^ ± 0.21
(0.18/0.43)	−	1.37^***^ ± 0.05
(0.09/0.21)	−	1.37^*^ ± 0.14
(0.04/0.11)	−	1.42^*^ ± 0.14
(0.02/0.055)	−	0.79^*^ ± 0.04
Verapamil (15/30)	1.24 ± 0.49	13.88^*^ ± 4.79
(0.054/0.11)	−	1.34^***^ ± 0.06
(0.027/0.055)	−	1.17 ± 0.17

*Reversal Fold (RF) values were calculated as IC_50_ of DOX alone/IC_50_ of DOX in the presence of the tested compounds. Statistical comparisons between IC_50_ of DOX alone or with compound in each treatment were performed by one-tailed paired t-test. Results represent the mean ± SE*.

*** *p < 0.001*,

** p < 0.01 and

* *p < 0.05*.

We also studied the reversal property of **14** to VLB resistance in Lucena 1 [VLB IC_50_ values of 2.49 μM (0.34–18.20) and 0.24 μM (0.04–1.89) in Lucena 1 and K562, respectively]. When **14** was co-administered with VLB, it significantly restored the sensitivity of the MDR cell line to VLB with a reversal fold activity of 60.20 (*p* < 0.0001), while the RF value in K562 was 9.63 (*p* < 0.05). Verapamil induced a decrease in the VLB IC_50_ of 49.17 (*p* < 0.0001) and 2.35-fold (*p* < 0.05) in Lucena 1 and K562, respectively.

### Doxorubicin intracellular accumulation assay

Based on the results of the reversal assay, we evaluated the capacity of **14** to inhibit the function of P-gp by measuring the intracellular accumulation of DOX by flow cytometry.

**Figure 4:**
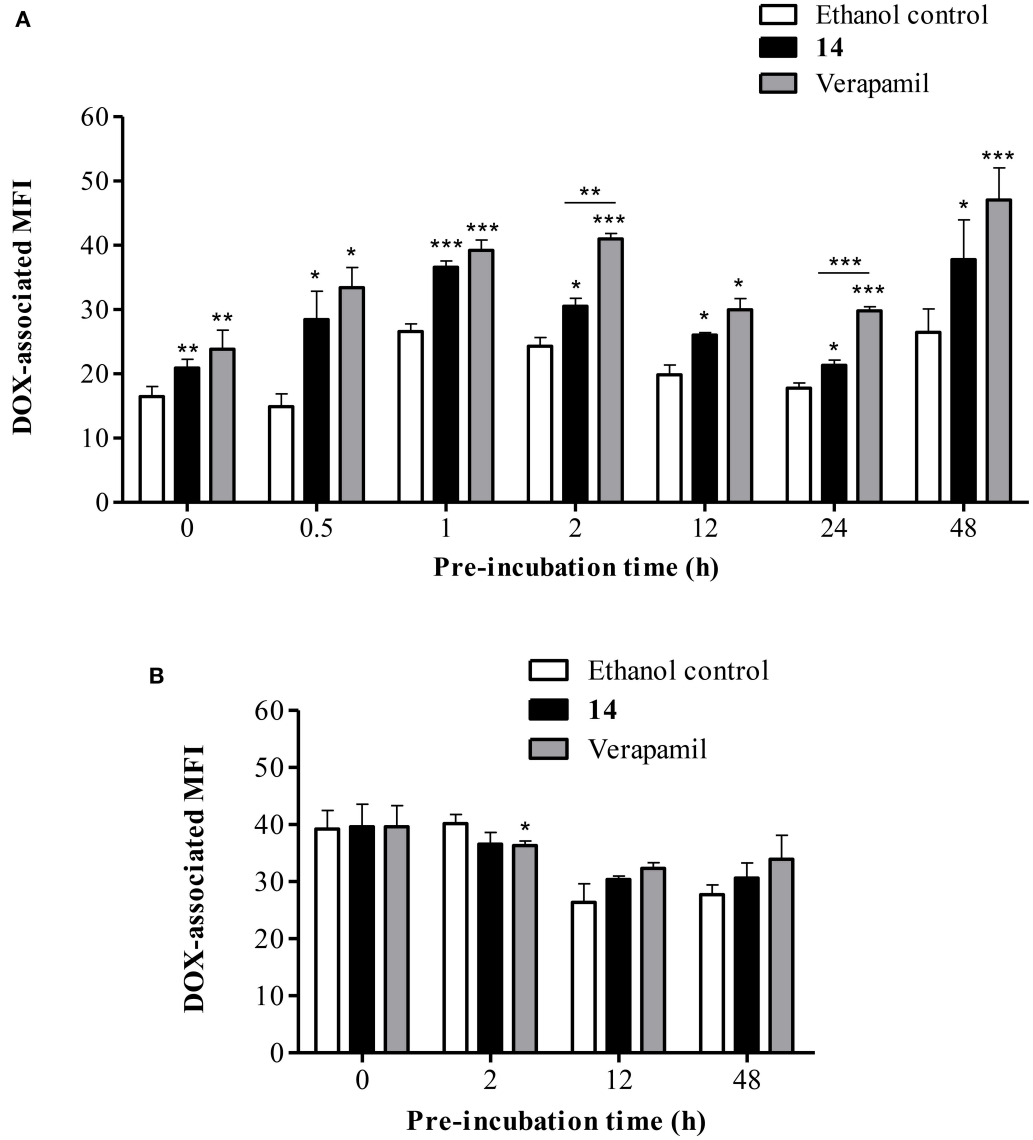
**Flow cytometric analysis of the effect of pinoresinol (14) on the intracellular accumulation of doxorubicin (DOX). (A)** Lucena 1 and **(B)** K562 cells were pre-incubated at different times with medium containing **14** at 112 μM before 1 h exposure to DOX. Each bar represents the mean ± SE. Significant differences from the ethanol control were determined at each time by using unpaired one-tailed Student's *t*-test (^***^p < 0.001, ^**^p < 0.01, ^*^p < 0.05).

To determine the desirable period of incubation, the uptake profile of DOX was first investigated at different periods of pre-incubation with **14**. As seen in Figure 4A, a significant increase (*p* < 0.01) in DOX accumulation by a factor of 1.3-fold was observed in Lucena 1 cells in the absence of pre-incubation compared with ethanol control cells, which showed the same rate of accumulation as untreated cells. The increase in DOX-associated MFI remained till 48 h of pre-incubation achieving a 1.4-fold increase. These values compared favorably with the 1.4 and 1.8-fold increase observed with verapamil at 0 and 48 h, respectively (*p* > 0.05). The highest difference in DOX accumulation with respect to the negative control was observed at 1 h pre-incubation (*p* < 0.001), at which time the highest percentage of inhibition related to verapamil was obtained (79% inhibition). Given these results, the selected pre-incubation time for further analysis was set at 1 h. No such increase in MFI was observed in K562 cells treated with **14** or with verapamil (Figure 4B).

**Figure 5:**
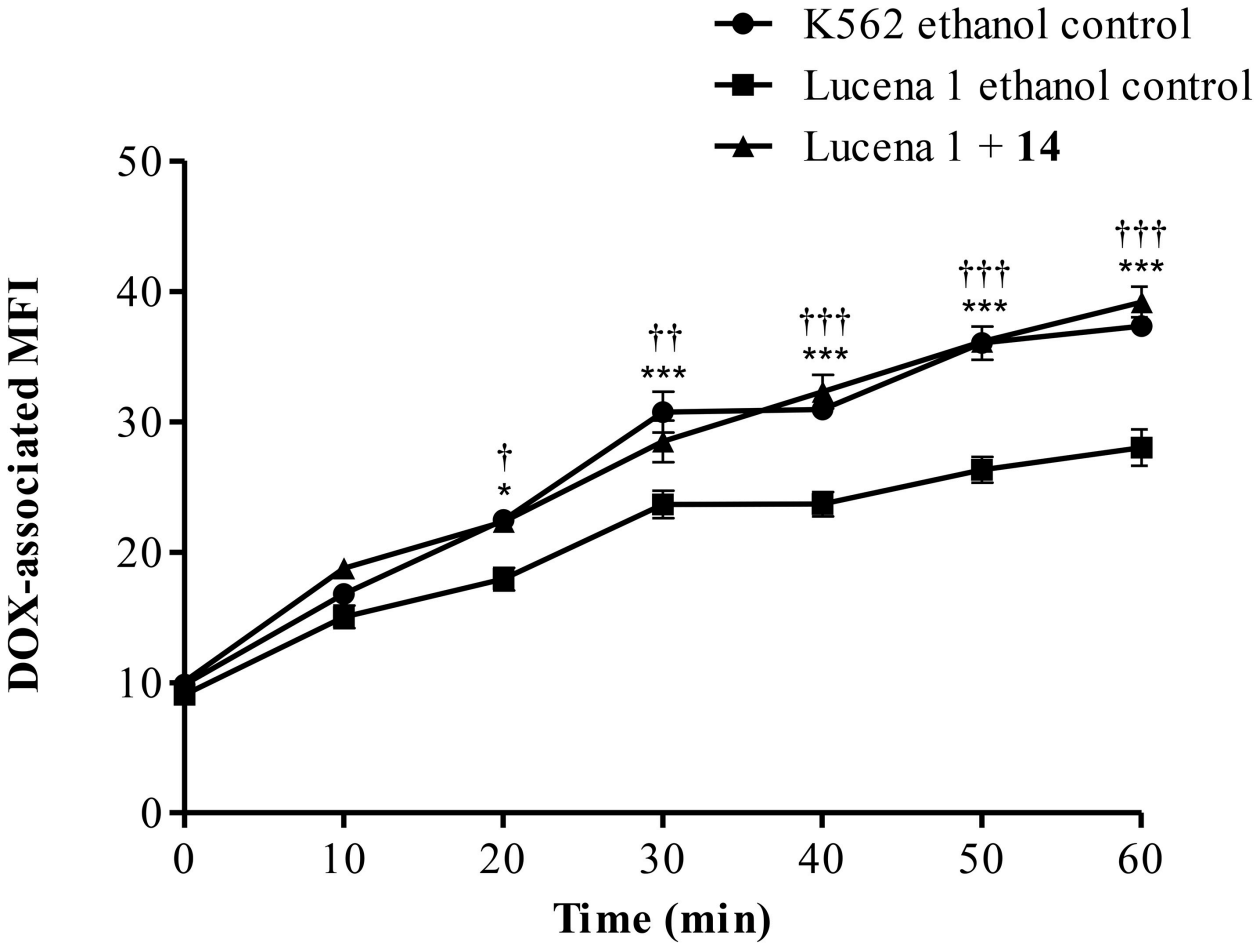
**Time course of doxorubicin (DOX) accumulation in Lucena 1 cells treated with pinoresinol (14) at 112 μM or ethanol (control) and in K562 cells treated with ethanol at different periods of time**. Data are expressed as mean ± SE. Significant differences at each time were determined by using two way analysis of variance (ANOVA) followed by the Bonferroni test (^***, †††^*p* < 0.001, ^††^*p* < 0.01, ^*, †^*p* < 0.05); ^†^: differences between Lucena 1 treated cells and Lucena 1 ethanol control and ^*^: differences between K562 ethanol control and Lucena 1 ethanol control.

The time course of DOX accumulation was further investigated. DOX- associated MFI increased in a time-dependent response in Lucena 1 treated with **14** with significantly higher values than that of Lucena 1 ethanol-treated cells (Figure 5). At 20 min, it was observed that **14** fully restored the presence of DOX within Lucena 1 cells (*p* < 0.05), reaching the MFI values observed in K562 (*p* > 0.05). This tendency was maintained over the whole period of time. The highest difference in MFI values in relation to the Lucena 1 ethanol control was observed at 1 h (Figure 5), determining that 1 h incubation with DOX was adequate.

**Figure 6:**
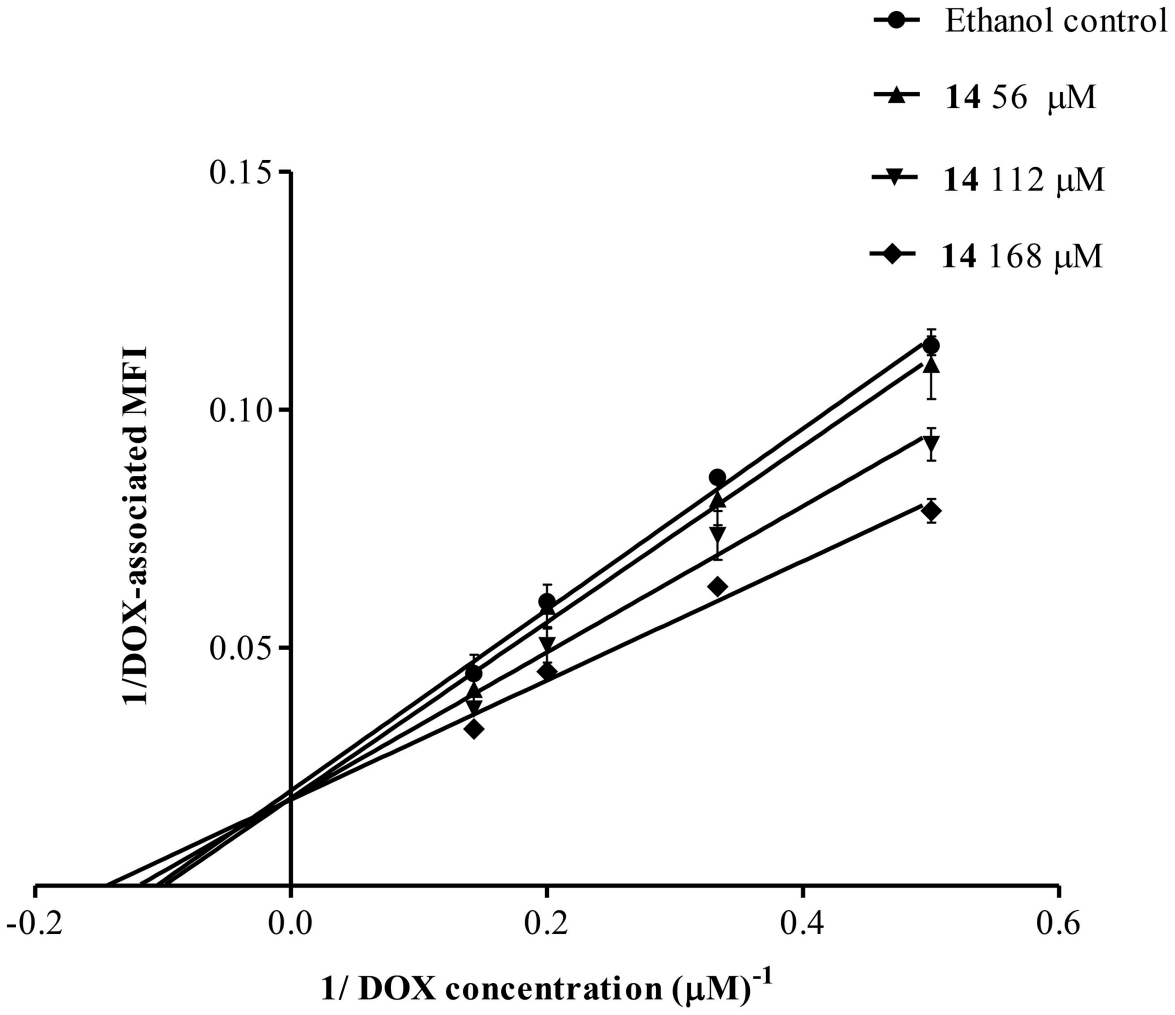
**Lineweaver-Burk double reciprocal plot for the kinetic analysis of pinoresinol (14)**. Lucena 1 cells were cultured for 1 h with a series of concentrations of **14** before 1 h exposure to DOX. The lines were drawn using linear least squares fit. Values are expressed as mean ± SE.

To further understand the mechanism of P-gp inhibition, we studied the behavior of **14**, monitoring the intracellular retention of DOX by flow cytometry. Increasing the concentrations of **14**, the values of MFI remained the same, while the Michaelis-Menten constant (*K*_m_) increased, as observed in the family of straight lines passing through the same point of the vertical axis (Figure 6). This plot indicated that **14** was a competitive inhibitor.

Following primary screening, **14** was assayed at serial dilutions to determine its minimum effective concentration (MEC). It significantly increased the intracellular accumulation of the substrate in Lucena 1 from 56 μM onwards (*p* < 0.05), with no differences with respect to verapamil (*p* > 0.05) (data not shown). No dose-dependency was observed. At the concentration mentioned and up to the maximum concentrations evaluated, no increase was observed in DOX accumulations in K562 (*p* > 0.05) (data not shown).

### Modulation of doxorubicin efflux

**Figure 7:**
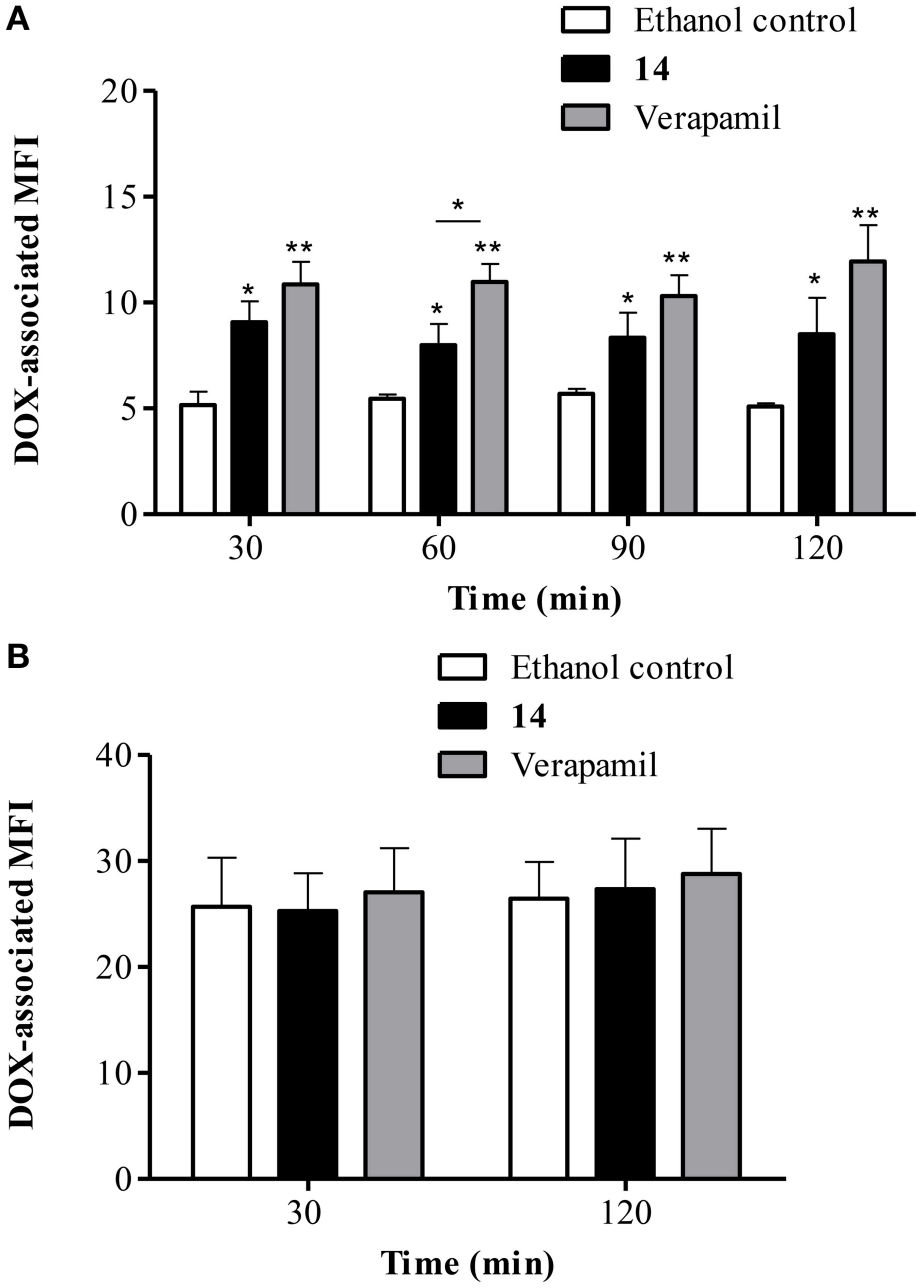
**Flow cytometric analysis of the effect of pinoresinol (14) on the efflux of doxorubicin (DOX) from (A)** Lucena 1 and **(B)** K562 cells. After 1 h pre-incubation with 14 at 112 μM, verapamil or ethanol, cells were exposed to DOX for 1 h. Subsequently, cells were washed and then incubated in the presence of **14** at 112 μM, verapamil or ethanol at various time points in a DOX-free medium. Data points represent the mean ± SE. Significant differences from the control were determined by using unpaired one-tailed Student's *t*-test (^**^p < 0.01, ^*^p < 0.05).

Since it was determined that **14** is capable of reversing DOX accumulation deficit, it was investigated whether this effect can be maintained after removing DOX from the medium. Figure 7A shows that **14** clearly increased DOX retention in Lucena 1 cells. At 30 min, the DOX-associated MFI increased 1.8-fold in comparison with control cells (*p* < 0.05) with similar activity to that observed with verapamil (*p* > 0.05). The effectiveness of **14** was maintained until the end of the experiment (Figure 7A). Neither **14** nor verapamil showed any significant effect on DOX efflux in K562 cells (Figure 7B).

**Figure 8:**
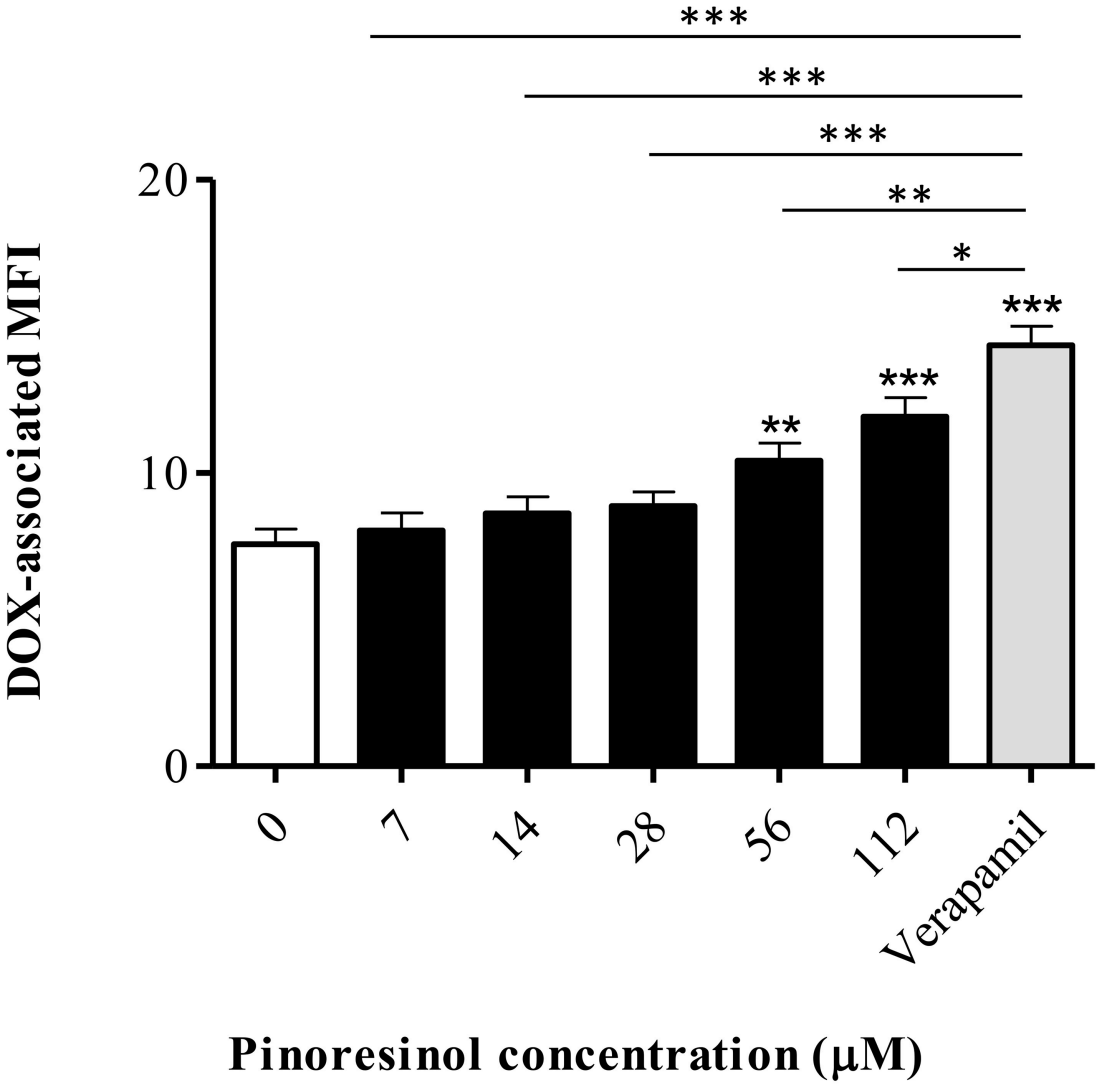
**Effect of a series of concentrations of pinoresinol (14) on the efflux of doxorubicin (DOX) in Lucena 1 cells**. Data points represent the mean ± SE. Significant differences from the control were determined by using unpaired one-tailed Student's *t*-test (^***^p < 0.001, ^**^p < 0.01, ^*^p < 0.05).

The minimum effective concentration (MEC) corresponded to 56 μM (*p* < 0.01), with significant differences with respect to verapamil (*p* < 0.01; Figure 8). A dose-dependent effect was observed (*b* = 0.037; *p* ≤ 0.0001; CI 95% = 0.023 to 0.051).

### Modulation of rhodamine 123 efflux

**Figure 9:**
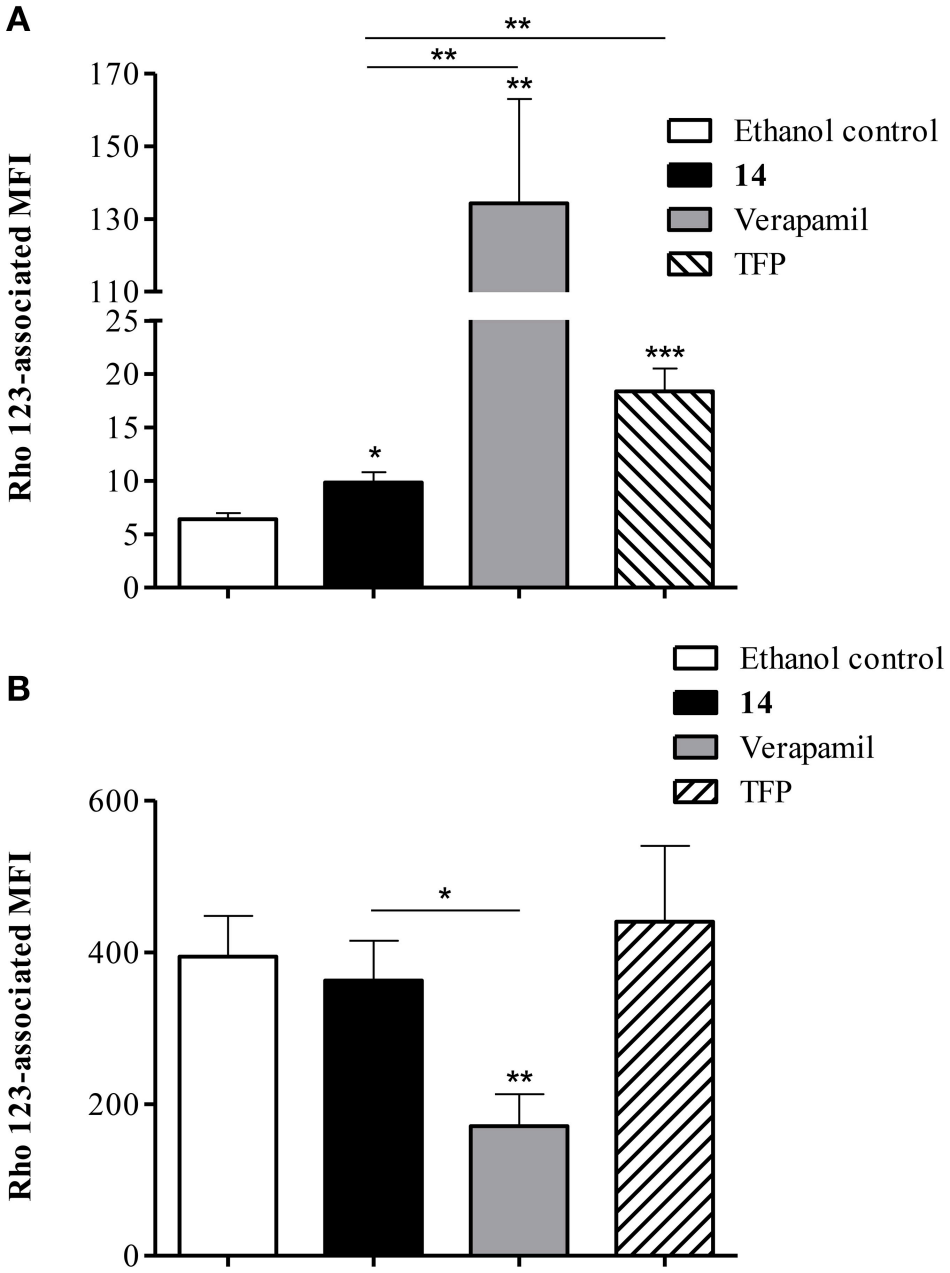
**Flow cytometric analysis of the effect of pinoresinol (14) on the efflux of rhodamine 123 (Rho 123) in (A)** Lucena 1 and **(B)** K562 cells. Both cell lines were pre-incubated with medium containing **14** at 112 μM, verapamil, trifluoperazine (TFP) or ethanol and then allowed to accumulate Rho 123 over 30 min. After washing, cells were incubated in probe-free medium in the presence of the assayed compounds for a further 30 min to allow Rho 123 extrusion. Each bar represents the mean ± SE. Significant differences from the control were determined by using unpaired one-tailed Student's *t*-test (^***^p < 0.001, ^**^p < 0.01, ^*^p < 0.05).

The ability of compound **14** to prevent the extrusion of another P-gp substrate, Rho 123, was further evaluated by flow cytometry. As shown in Figure 9A, exposure of Lucena 1 cells to **14** enhanced intracellular Rho 123 intensity 1.5-fold. This effect was significantly different from that observed with verapamil and with TFP, another known P-gp inhibitor (*p* < 0.01), but to a lesser extent with the latter. Compound **14** did not induce any increase in Rho 123-associated MFI in K562 (Figure 9B).

### Determination of the ATPase activity

**Figure 10:**
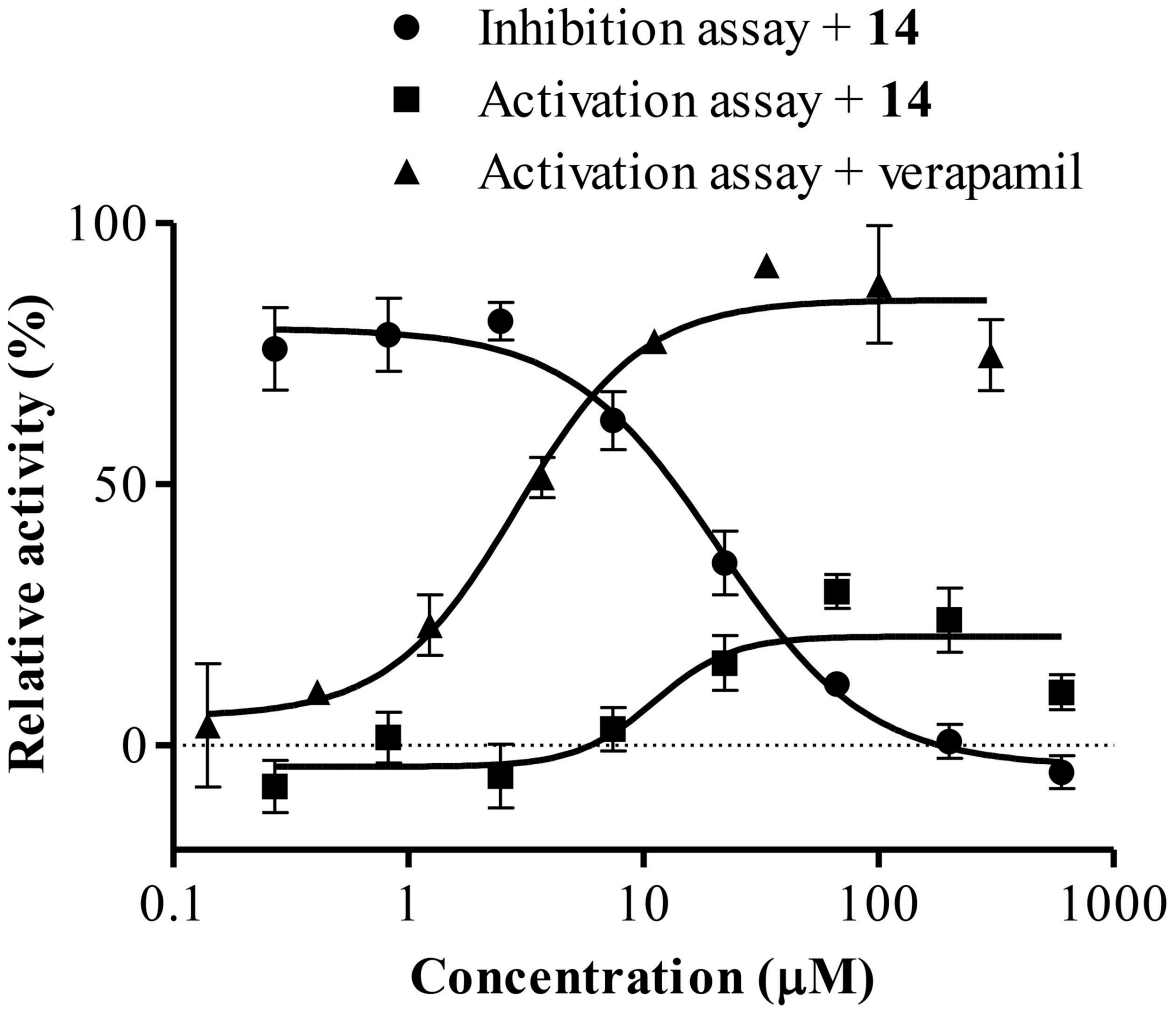
**Effect of 14 on ATPase activation and inhibition**. Data points represent the mean ± SE.

To further characterize the P-gp-resistance reversal properties of **14**, its influence on basal and verapamil-stimulated ATPase activity was determined. As observed in Figure 10, compound **14** mildly stimulated P-gp-ATPase activity with a maximum of 29.49 ± 4.55% at 66.7 μM, a lower value than that observed with verapamil. It was also observed that **14** antagonized the verapamil-stimulated ATP hydrolysis with an IC_50_ of 20.89 μM (17.39–23.61).

It is worth mentioning that **14** had no effect on the ATPase activity of control membranes up to 600 μM and that a complete inhibition of verapamil-stimulated ATPase hydrolysis was observed with CsA (data not shown).

### Molecular modeling

**Figure 11:**
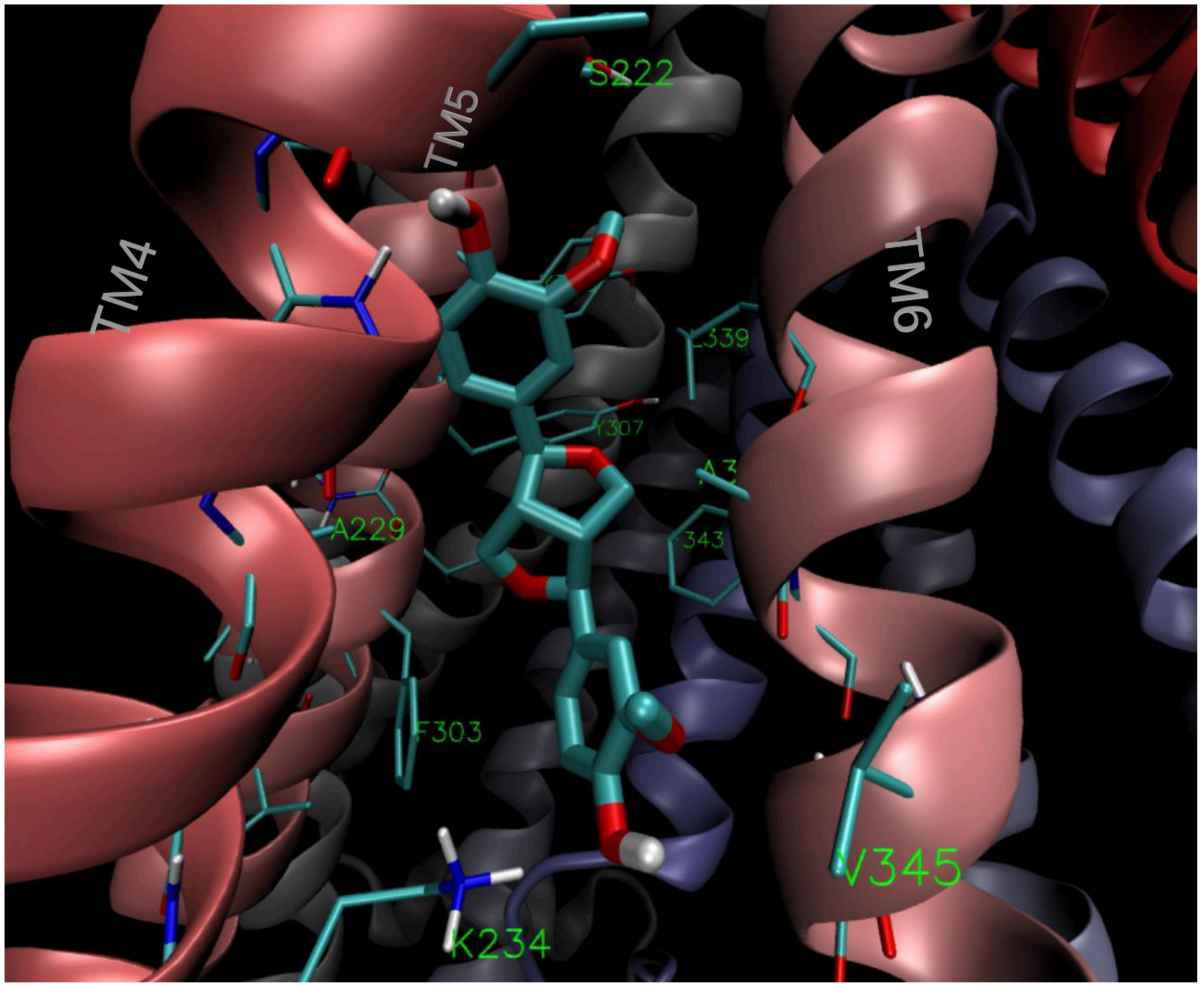
**Binding mode of the lowest energy conformation of (+)-pinoresinol (14) showing the main contacts of 14 with residues from TMH 4, 5, and 6**.

The estimated free energy of binding and the corresponding inhibition constants obtained for the (+)- and (−)-pinoresinol (**14**) are summarized in Table 2, together with the values obtained for the model substrate DOX, compound **2**, as the negative control and the mild and powerful known inhibitors verapamil and tariquidar. As observed, there is a small difference in the estimated binding energies (0.14 kcal/mol) between (+)- and (−)-pinoresinol. Both enantiomers went to the same site, with slight differences in the pose. The binding mode of the subject compound **14** is shown on Figure 11, revealing as main contacts S222, A223, K234, F303, Y307, V310, L339, and F343, most of which are residues from TMH 4, 5 and 6. Almost all of these are also involved in binding the reference inhibitor verapamil and part of them also to the powerful inhibitor tariquidar. The K_I_ obtained for tariquidar in the sub-nM order (Table 2) is close to that experimentally found [58], while the value obtained for the negative control, **2** is almost 100 μM, which is inactive in practice, matching that observed experimentally.

**Table 2:** **Brief summary of docking results**.

**Compounds**	**Estimated free energy of binding (kcal/mol)**	**Inhibition constant K_I_ (nM)**
DOX	−8.13	1100
Rho 123	−8.08	1200
2	−5.66	71580
16	−9.17	191
16a	−8.72	402
1-hydroxy-(+)-pinoresinol	−8.59	505
Phylligenin	−8.84	338
14 (+)-pinoresinol	−8.53	540
14 (-)-pinoresinol	−8.39	711
pinoresinol diglucoside	−8.26	881
pinoresinol 4-glucoside	−8.55	540
Verapamil	−9.01	249
Tariquidar	−12.36	0.32

On the other hand, it was observed that the affinity of **14** for the NBD region was substantially lower than that for the region in the TMD.

As also observed in Table 2, compound **16** was found to have the most favorable binding energy among the set of pinoresinol derivatives that were evaluated with the intention of improving the activity of **14**. Compound **16** was also found to be 0.45 kcal/mol more favored than its own enantiomer **16a** [derivative of the (−)-pinoresinol], showing a still mild but clearer stereospecificity than for the case of **14**. This result encouraged the further experimental study of compound **16**.

**Figure 12:**
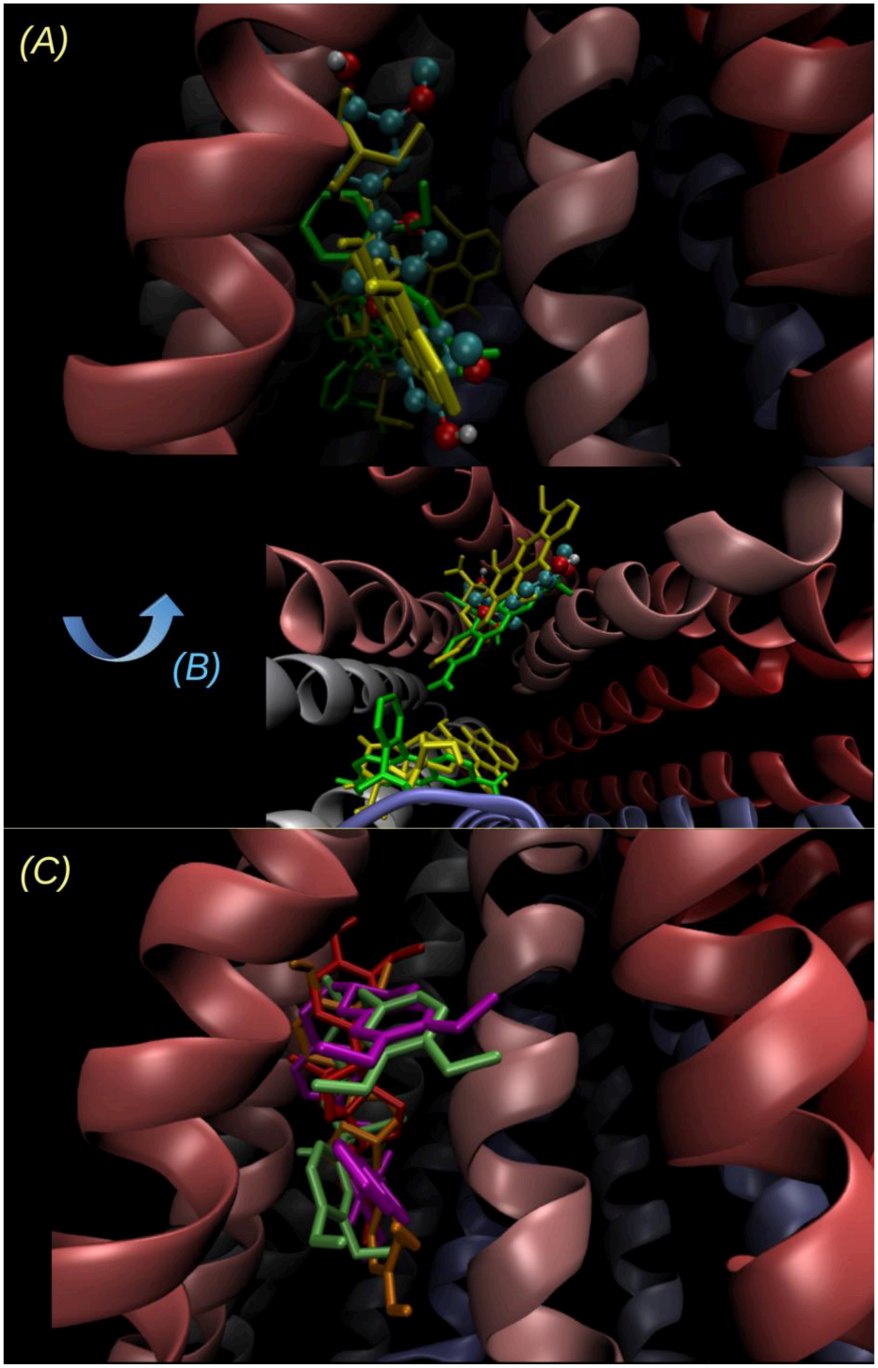
**Two different views of the lowest energy docked structure of 14. (A)** Point of view looking to the inverted “V” within the bilayer plane, the same point of view as in Figure 11 and **(B)** looking from the intracellular side to outside (perpendicular to the bilayer plane). The binding pose of **14** (ball and sticks) is compared to the main and secondary sites of Rhodamine 123 (yellow tubes) and DOX (green tubes). The sites of lowest energy are opposite for Rho 123 and DOX, and the energy difference between the secondary site and the main (reported on Table 2) is below 0.5 kcal/mol in both cases. **(C)** Superimposition of the lowest energy poses of **14** (orange), **16** (red), and the known inhibitors verapamil (lime) and tariquidar (violet).

The poses of **14**, **16** and tariquidar are superimposed on Figure 12C. Compounds **14** and **16** overlap with the binding region of tariquidar in one of the homolog halves at the top of the inverted “V” formed by the TMHs. This region overlaps with a secondary DOX binding site (0.5 kcal/mol less stable than the main site i.e., the one with the more negative binding energy, reported in Table 2; see also Figures 12A–C). As previously discussed (Jara et al., 2013), the binding of the subject compound seems to be just the first step of a complex process involving long range conformational changes induced in the transporter. In this context, the fact of finding coincidences in the main contacts compared to those observed for known powerful inhibitors would be a promising feature, besides the binding energies themselves.

### Study on the surface expression of P-Gp

**Figure 13:**
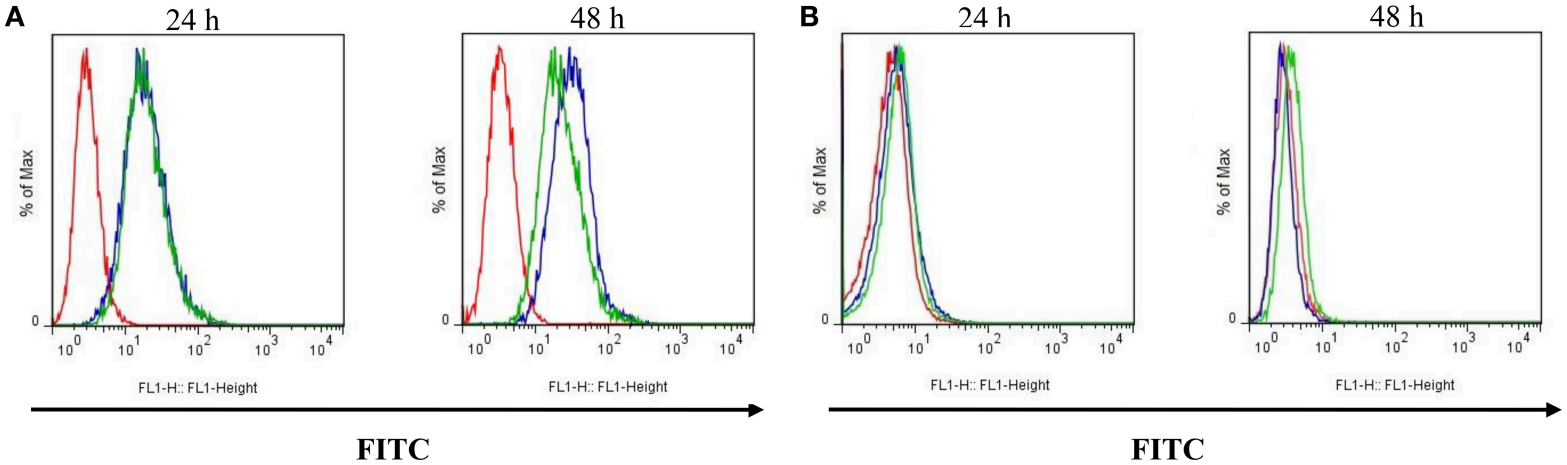
**Effect of pinoresinol (14) on the surface expression of P-glycoprotein by flow cytometric analysis. (A)** Lucena 1 and **(B)** K562 cells were stained with FITC-labeled mouse anti-human P-glycoprotein antibody at 24 and 48 h. Unstained cells are shown with red histogram, stained cells with blue histogram and cells stained and treated with **14** at 112 μM are shown with green histogram. Histograms are representative of three independent experiments.

P-gp expression at the membrane surface was lower in Lucena 1 cells treated with **14** compared to the control group measured at 48 h (Figure 13A).

No suppression of P-gp presence was observed in the outer membrane in K562 cells at the study times (Figure 13B).

### *In vitro* activity of 1-acetoxy-(+)-pinoresinol (16)

The pinoresinol derivative **16**, which showed the most promising activity *in silico* (in terms of binding energy, Table 2), showed an increase in DOX cytotoxicity by a factor of 22.7 at 14 μM and 1.4 at 0.11 μM (Table 1). There was no significant difference in relation to verapamil at 30 and 0.11 μM with **16** applied at 14 and 0.11 μM, respectively (*p* > 0.05). At 14 μM, no potentiation was observed in DOX activity in K562 cells (*p* > 0.05). When tested at the maximum non-toxic concentration (28 μM), it caused a 96.8-fold sensitization to DOX in Lucena 1, while a 17.3-fold activity was observed in K562, meaning that the shift in the IC_50_ of DOX was not only due to P-gp inhibition. For this reason, this concentration was not used in further assays.

**Figure 14:**
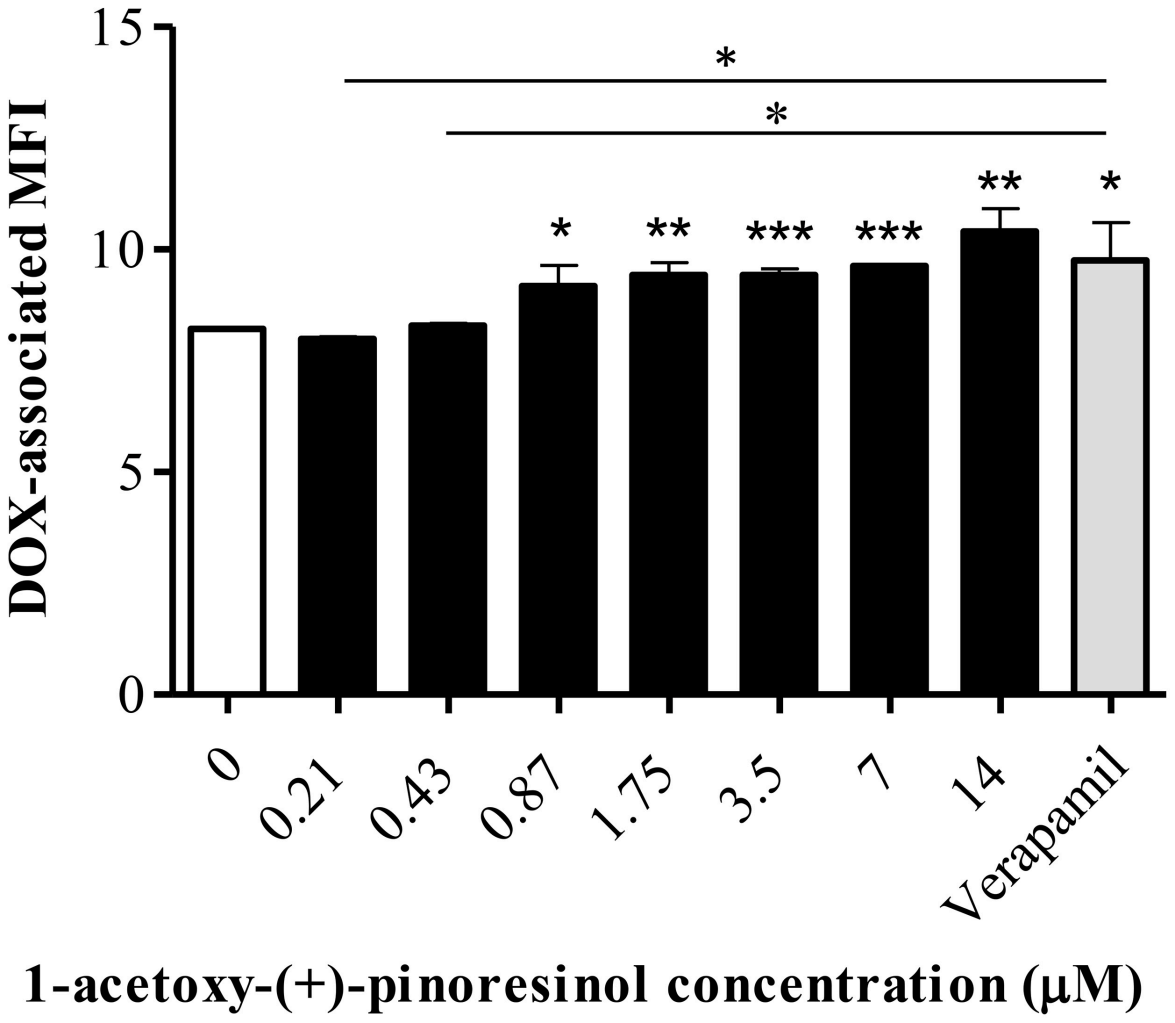
**Effect of a series of concentrations of 1-acetoxy-(+)-pinoresinol (16) on the accumulation of doxorubicin (DOX) in Lucena 1 cells**. Significant differences from the control were determined by using unpaired one-tailed Student's *t*-test (^***^p < 0.001, ^**^p < 0.01, ^*^p < 0.05).

In the accumulation assay, **16** significantly increased the DOX-associated MFI in Lucena 1 from 0.87 μM (*p* < 0.05) with no difference with respect to verapamil (*p* > 0.05; Figure 14). As also occurred with **14**, no dose-dependency and no increase in DOX accumulation were observed in K562 (*p* > 0.05) (data not shown) at the MEC and up to the maximum concentration evaluated.

### Cytotoxicity on peripheral blood mononuclear cells (PBMC)

To determine whether **14** or **16** were toxic on non-tumoral human cells, PBMC were obtained. These cells were considered the most adequate in relation to the type of tumor cells evaluated in this study. An MTT assay showed that **14** and **16** displayed IC_50_ values of 216.25 μM (125.93–371.35) and 102.57 μM (48.60–216.45), respectively.

## Discussion

P-gp modulation is gaining more and more ground in drug discovery due to the clinical importance of this pump owing to its association with MDR (Loo and Clarke, 1999). With the aim of finding novel inhibitors of P-gp function, we screened 15 bioactive plant-derived principles obtained in the laboratory. Compound **14** (Figure 1) showed the highest effectiveness in the reversal assay, potentiating DOX cytotoxicity in Lucena 1 cells from 7 μM (Table 1). When applied at 112, 28 and 14 μM, it showed a comparable effect in circumventing the MDR phenotype to that of verapamil at 30 μM (*p* > 0.05). When the combination of **14** and DOX was evaluated on the sensitive K562 cell line, no decrease was seen in the inhibitory value of the chemotherapeutic drug, and thus any other interaction between these compounds different from P-gp inhibition was discarded. It was remarkable that compounds **8** and **10** did not cause any effect in DOX toxicity, while **11** decreased DOX sensitivity (*p* < 0.01). Although these compounds have been described as P-gp inhibitors (Deferme and Augustijns, 2003; Ofer et al., 2005; Borska et al., 2012; Saeed M et al., 2015), in some assays no activity or stimulatory activity was found (Deferme and Augustijns, 2003; Khantamat et al., 2004; Katayama et al., 2007; Wu et al., 2016), which agrees with our findings. These contradictory observations have been previously reported by other authors (Di Pietro et al., 2002; Deferme and Augustijns, 2003) and could be due to the different concentrations used in the experiments (Yuan et al., 2012), the type of cancer cells tested (Di Pietro et al., 2002) and differences in the interactions between the flavonoids and P-gp depending on the substrate used in the experiments (Di Pietro et al., 2002; Eid et al., 2015). On the other hand, the chalcone **6** was capable of potentiating DOX toxicity, not only by interfering with P-gp, as was previously determined (Chieli et al., 2012), but also and mainly, by some additional mechanism as deduced by the RF observed in K562 (Table 1).

The effective compound **14** also potentiated the cytotoxicity of VBL in Lucena 1 cells (*RF* = 60) and in K562 to a lesser extent (*RF* = 9). The RF of **14** was comparable to that of verapamil (*RF* = 49) (*p* > 0.05), which also decreased VLB IC_50_ in K562 (*RF* = 2). These results showed that, besides the inhibition on P-gp mediated transport, which seems predominant, **14** and verapamil also induce another kind of interaction. It was previously reported that verapamil enhanced the cytotoxicity of VLB not only in the resistant P388/VCR cell line but also in the sensitive cell line P388 (Tsuruo et al., 1981).

The activity demonstrated by **14** in the MTT assay was confirmed by examining the intracellular accumulation of DOX, which specifically reflects the reversion of drug transport out of cells. As seen in Figure 4A, compound **14** showed a clear increase in intracellular DOX-associated fluorescence in the absence of pre-incubation, an effect that remained till the end of the study. Huang et al. (2013) established that compounds with an activity >30% of the maximal activity exerted by verapamil were considered as P-gp inhibitors. In our results, **14** showed inhibitory activity throughout the course of the study (inhibition at 0 and 48 h = 60 and 55%, respectively), with a maximum effect at 0.5 and 1 h of experiment (73 and 79% of inhibition, respectively). These values showed that the activity of **14** is particularly noteworthy, and the long-term effectiveness suggested that the lignan was not inactivated by cellular metabolism. It is important to note that a decrease in the superficial expression of the pump was observed at 48 h, meaning that at this time a sum of events could influence the results. On the other hand, **14** showed no effect on DOX accumulation when tested in K562 cells (Figure 4B). This strongly supports the proposition that **14** reverses the efflux of DOX out of the cells specifically by interfering with P-gp function. It has been previously described that (+)-pinoresinol increased the intracellular accumulation of calcein-AM when applied at 50 and 100 μM (Tamaki et al., 2008). However, no additional information about the P-gp-mediated resistance-reversing effect of this compound has been reported and there is no evidence showing the mechanism underlying this property. As far as we know, there are no reports evaluating the racemic mixture.

The time course study showed that **14** was effective at retaining DOX in Lucena 1 cells at 20 min from the beginning (*p* < 0.05; Figure 5), showing that short-term treatment with the modulator leads to effectiveness. The fact that the intracellular DOX fluorescence intensities obtained in Lucena 1 treated with **14** were similar to those obtained in K562 (*p* > 0.05) showed the potency of **14** for fully blocking the efflux of the drug.

To obtain further information about the type of inhibition exerted by **14**, the kinetic behavior of DOX accumulation was analyzed by the Lineweaver-Burk double reciprocal method (Figure 6), which showed that the lignan was a competitive inhibitor of P-gp.

The addition of **14** from 56 μM significantly enhanced cellular DOX accumulation compared to control cells with an 80% inhibition with respect to verapamil. This MEC differs from that obtained in the resistance reversal assay, in which it was observed that **14** was active from 7 μM (Table 1). This discrepancy, also reported by other authors (Schuurhuis et al., 1989; Lan et al., 1996), seems not to be related to the different incubation periods or DOX concentrations used in both experiments, since it was previously demonstrated that these parameters did not influence the results (Pereira and Garnier-Suillerot, 1994; Lan et al., 1996). Lan et al. (1996) explained this discrepancy stating that the amplitude of the reversion of DOX resistance exerted by a compound was higher than the increase of its intracellular accumulation. This difference would be supported by previous findings regarding subcellular DOX localization (Schuurhuis et al., 1989, 1993; Pereira and Garnier-Suillerot, 1994; Lan et al., 1996). In the accumulation assays, flow cytometry measures all the DOX inside the cell, including that sequestered inside cytoplasmic vesicles attributable to the pumping of the drug into them by P-gp (Lan et al., 1996; Munteanu et al., 2006; Yu et al., 2008), while the phenomenon of cell killing is only influenced by the presence of DOX in the nucleus (Yu et al., 2008). In the absence of **14**, a small concentration of the cytotoxic agent trapped in the vesicles will be considered in the measurements of fluorescence, resulting in a lower difference in comparison to the treatment. The possible effect of **14** in blocking both plasma and vesicular membrane P-gp would lead to an increase of DOX in the nucleus, as is also observed with verapamil (Schuurhuis et al., 1993). In this situation, the change in DOX toxicity will be greater. These observations indicated that **14** increased not only DOX accumulation but also its toxicity.

Although a dose- response relationship was not clearly seen in the accumulation assay, this could be seen in the reversal and efflux assays, indicating that the behavior of compound **14** was dose-dependent. Similar results were previously obtained with some thioxanthones for the reversal and accumulation assays (Palmeira et al., 2012b).

The results obtained in the efflux assays showed that compound **14** increased the accumulation of DOX to a slightly higher extent than that of Rho 123 [retention increased 1.8 (Figure 7A) and 1.5 times (Figure 9A), respectively at 30 min of experiment]. This suggested that **14** is a P-gp inhibitor which shows a different level of potency depending on the substrate used. Rho 123 binds to the R site (Loo and Clarke, 2014), while the binding site for DOX is predominantly located at the H-site (Zeino et al., 2015). Overlapping but distinct drug specificities of these sites (Shapiro and Ling, 1997) would explain the different level of effectiveness exerted by **14**. Docking studies (Figure 12C) showed that **14**, **16** and tariquidar bind to P-gp in the H binding site, located on TMH 4, 6, 10, 11, and 12 (Gutmann et al., 2010), agreeing with previous findings reported for tariquidar (Li et al., 2015). However, due to the similarity in the binding energies of both substrates and the small differences in the affinities of the main and secondary sites, the docking results seem not to shed light on this issue.

It has been previously reported that other lignans, such as schisandrin A (Huang et al., 2008; Xia et al., 2015), schisandrin B (Qiangrong et al., 2005; Huang et al., 2008), schizandrol A (Huang et al., 2008), nirtetralin, niranthin, phyllanthin, and phyltetralin (Leite et al., 2006), arctigenin, arctiin, matairesinol, (iso)lappaol A, lappaol C and lappaol F (Nabekura et al., 2008; Su et al., 2015) showed an inhibitory effect against P-gp activity. It is worth noting that the closely related lignan (-)-sesamin increased the accumulation of daunorubicin from a concentration of 50 μM in KB-C2 cells overexpressing P-gp (Nabekura et al., 2008).

To gain deeper insight into the characteristics of the reversal activity of **14** on the functional aspect of P-gp, its effects on P-gp-ATPase activity were investigated as well as its binding mode to the glycoprotein. Examining the effects of **14** on P-gp ATPase, it was observed that it slightly activated the basal ATP hydrolysis and antagonized verapamil-stimulated ATPase activity (Figure 10). The latter effect arose from docking analysis.

*In silico* analysis determined that **14** binds to P-gp in the apex of the V-shaped transmembrane barrels, a site proposed as key for substrate/inhibitor recognition. As seen in Figure 11, **14** binds to the aromatic aminoacids located in TMH 4, 5 and 6. This region also found for **16**, clearly overlaps with that found for verapamil and for contacts of tariquidar with one of the homolog halves of the TMB (Figure 12). Most of the residues involved in the binding of **14** were also contacts of this last reference inhibitor. Most of these residues were proposed as relevant in the binding of inhibitors in experimental and computational bases, as previously discussed (Jara et al., 2013). These results would shed some light on the nature of its interaction with P-gp at molecular level and merit further mechanistic and kinetic studies.

The effect of **14** on the surface expression of P-gp was determined by flow cytometry. The experiments showed that treatments with **14** resulted in less P-gp at the Lucena 1 cell membrane at 48 h. These results would indicate that **14** countered drug resistance, not only by modulating P-gp function but also by interfering with the presence of P-gp in the cell surface, which also prevents drug efflux (Loo and Clarke, 1997, 2000).

Considering compound **14** as a promising lead compound, virtual derivatives with pinoresinol scaffold were submitted to molecular modeling studies. Based on the results obtained, compound **16** deserved further evaluation since its estimated free energy of binding (−9.17 kcal/mol) was similar to or even better than that of verapamil (−9.01 kcal/mol). The result showed that **16** was 64 times more effective than **14** according to the MECs obtained by the reversal (0.11 and 7 μM, respectively) and the accumulation assays (0.87 and 56 μM, respectively), thus demonstrating outstandingly improved activity. The same explanation of the discrepancy in the MECs of **14** obtained by the reversal and accumulation assays could also be applied to explain the difference obtained with **16**. At the minimum concentration that increased intracellular DOX, **16** showed 62% activity compared to verapamil while it showed superior activity to the reference compound (141%) when cells were exposed to 14 μM (Figure 14). The absence of an increase in DOX accumulation in K562 indicated that **16** specifically blocked efflux mediated by P-gp. Further analyses are needed to characterize the activity and the mechanism of action of **16** in more detail.

The IC_50_ of 216.2 and 102.6 μM displayed by **14** and **16**, respectively, on PBMC indicated an absence of cytotoxicity, based on the statement of the US National Cancer Institute plant screening program which established as cytotoxic those pure compounds with mean toxicity values below 10 μM (Kuete et al., 2014). It is important to note that the IC_50_ values obtained were much higher than the effective concentrations observed for both lignans. In addition to the presence of **14** in *Melia azedarach* (Carpinella et al., 2003), this compound and also **16** are present, in considerable proportions, in virgin olive oil which is an important component of the diet (Fini et al., 2008; López-Biedma et al., 2016). A case-control study of the protective role of pinoresinol in breast cancer risk demonstrated that the median daily consumption of **14** was 60 μg (Torres-Sanchez et al., 2009).

## Conclusion

Our findings demonstrated that compound **14** and its derivative **16** are likely to be promising P-gp chemosensitizers. This property, the low cytotoxicity demonstrated by these lignans and its presence in the daily diet, position them as potential candidates for developing compounds to use in combination with anticancer drugs, which could lead to significant advances in leukemia therapies.

## Author contributions

MC contributed to the experimental design and was in charge of setting up all the assays and the writing of the manuscript. DV contributed to the experimental design and was in charge of the molecular modeling studies. MG, JL, and MJ conducted the experiments, while PL and SG performed the *in silico* determinations. MM and SP advised on the experimental aspects and on writing, respectively. GM advised on the statistical analysis. VR contributed to the experimental design and the writing of the manuscript. All authors contributed to revising the draft and approved the final version of the paper.

## Funding

This research project was conducted with the support from Roemmers Foundation, Catholic University of Córdoba, MINCyT Córdoba (GRF 2008; PID 2012 Call 2013) CONICET (PIP 11220100100236), PICT 2014-1594 and FAPERJ-CONICET (E-26/110.059/2014-Argentina. Programa Edital 26/2013). MG, JL, and MJ acknowledge receipt of a Scholarship from CONICET.

### Conflict of interest statement

The authors declare that the research was conducted in the absence of any commercial or financial relationships that could be construed as a potential conflict of interest.

## Abbreviations

P-gp: P-glycoprotein
MDR: multidrug resistance
TMD: transmembrane domains
TMH: transmembrane α-helices
CML: chronic myelogenous leukemia
MTT: 3-(4,5-Dimethyl-2-thiazolyl)-2,5-diphenyl-2H-tetrazolium bromide
VLB: vinblastine sulfate
CsA: cyclosporine A
TFP: trifluoperazine
DOX: doxorubicin
MEC: minimum effective concentration
Rho 123: rhodamine 123
NBD: nucleotide-binding domain
PBMC: peripheral blood mononuclear cells.
